# Increased virulence of *Puccinia coronata f. sp.avenae* populations through allele frequency changes at multiple putative *Avr* loci

**DOI:** 10.1371/journal.pgen.1009291

**Published:** 2020-12-28

**Authors:** Marisa E. Miller, Eric S. Nazareno, Susan M. Rottschaefer, Jakob Riddle, Danilo Dos Santos Pereira, Feng Li, Hoa Nguyen-Phuc, Eva C. Henningsen, Antoine Persoons, Diane G. O. Saunders, Eva Stukenbrock, Peter N. Dodds, Shahryar F. Kianian, Melania Figueroa

**Affiliations:** 1 Department of Plant Pathology, University of Minnesota, St. Paul, Minnesota, United States of America; 2 USDA-ARS Cereal Disease Laboratory, St. Paul, Minnesota, United States of America; 3 Environmental Genomics Group, Max Planck Institute for Evolutionary Biology, Plon, Germany; 4 Christian-Albrechts University of Kiel, Kiel Germany; 5 INRA/Universite de Lorraine Interactions Abres/Microorganismes, Champenoux, France; 6 John Innes Centre, Norwich, United Kingdom; 7 Commonwealth Scientific and Industrial Research Organisation, Agriculture and Food, Canberra, Australia; INRA, FRANCE

## Abstract

Pathogen populations are expected to evolve virulence traits in response to resistance deployed in agricultural settings. However, few temporal datasets have been available to characterize this process at the population level. Here, we examined two temporally separated populations of *Puccinia coronata* f. sp. *avenae* (*Pca*), which causes crown rust disease in oat (*Avena sativa*) sampled from 1990 to 2015. We show that a substantial increase in virulence occurred from 1990 to 2015 and this was associated with a genetic differentiation between populations detected by genome-wide sequencing. We found strong evidence for genetic recombination in these populations, showing the importance of the alternate host in generating genotypic variation through sexual reproduction. However, asexual expansion of some clonal lineages was also observed within years. Genome-wide association analysis identified seven *Avr* loci associated with virulence towards fifteen *Pc* resistance genes in oat and suggests that some groups of *Pc* genes recognize the same pathogen effectors. The temporal shift in virulence patterns in the *Pca* populations between 1990 and 2015 is associated with changes in allele frequency in these genomic regions. Nucleotide diversity patterns at a single *Avr* locus corresponding to *Pc38*, *Pc39*, *Pc55*, *Pc63*, *Pc70*, and *Pc71* showed evidence of a selective sweep associated with the shift to virulence towards these resistance genes in all 2015 collected isolates.

## Author summary

The rust fungus *Puccinia coronata* f. sp. *avenae* (*Pca*), which causes crown rust disease, decimates oat (*Avena sativa*) production in many countries of the world. While the use of genetic resistance in crop breeding programs is the most sustainable disease management strategy to control plant disease, the release of oat varieties that display genetic resistance to *Pca* infection is hindered by rapid evolution of this pathogen. This study aims to determine demography and determinants of adaptive evolution in *Pca* to minimize the risk of disease outbreaks and enhance resistance gene stewardship. We recently published two high quality genome references of *P*. *coronata* f. sp. *avenae*. Here, we used these resources to direct a population genomics-based study of two temporally distant sets of pathogen collections to study genotypic changes that may explain the most recent oat crown rust epidemics across the continental US. We found that the population of *Pca* in 1990 is significantly different to that collected in 2015 at both genotypic and phenotypic levels. Our findings are consistent with the role of sexual and asexual reproduction in the *Pca* population diversity. Importantly, our work identifies genomic regions and genes that may be involved in local host adaptation which in the future may assist in the development of molecular markers and diagnosis of virulence.

## Introduction

Plant disease resistance is often mediated by the recognition of pathogens via immune receptors, which then activate defense responses that prevent pathogen growth [1]. This pathogen recognition system was initially described in the gene-for-gene model, in which dominant resistance (*R*) genes in the host plant confer recognition of specific avirulence (*Avr*) genes from the pathogen [2]. *R* genes generally encode intracellular immune receptor proteins of the nucleotide binding leucine rich repeat (NB-LRR) class, which recognize pathogen ‘effector’ proteins that are delivered into host cells during infection to suppress host basal defenses and facilitate infection [1,3]. Consequently, plants and pathogens engage in a co-evolutionary battle, where new resistance traits in the host are countered by gain-of-virulence traits in the pathogen. Studies in wild host-pathogen populations suggest that this antagonistic co-evolution maintains a balance between variation in host resistance and pathogen virulence [4]. However, in agroecosystems, breeding for resistance based on NB-LRR-encoding genes [5] and subsequent monoculture deployment creates an environment that promotes directed evolution of the pathogen and the rapid emergence of new virulence traits in pathogen populations [6]. There have been few population level studies examining the processes involved in virulence evolution in agricultural pathogens over time.

Rust fungi are pathogens of many plants [7] and the continual emergence of new virulent strains of rust represents serious threats to the production of small grains and global food security [8]. Cereal rust fungi propagate asexually in their primary host and rely on alternate hosts to complete their sexual reproduction cycle. Gain-of-virulence phenotypes can often appear as a result of mutations in asexual lineages of cereal rusts [9]. For instance, virulence to the resistance genes *Sr35* and *Sr50* in wheat resulted from insertion mutations in the corresponding *AvrSr35* and *AvrSr50* genes of the wheat stem rust pathogen *Puccinia graminis* f. sp. *tritici* (*Pgt*) [10,11]. These are the only two *Avr* genes identified in cereal rusts to date, so our broader understanding of virulence evolution is limited. Shuffling of virulence factors through sexual reproduction can also contribute to genetic diversity of fungi [12]. The prevalence of the alternate (sexual) host has been associated with increased intensity of rust epidemics historically, and accordingly barberry eradication in the United States of America (US) is credited with decreasing stem rust infection [13,14]. Somatic hybridization, as shown in the broadly virulent Ug99 lineage of *Pgt*, is another mechanism that contributes to genetic variation in rusts [15]. Thus, there are multiple processes that can play a role in the evolution of rust fungi and their precise contribution has not yet been determined.

*Puccinia coronata* f. sp. *avenae* (*Pca*), causes crown rust disease in oat (*Avena sativa*), and populations of this pathogen exhibit high variability in virulence phenotypes on oat varieties and rapid emergence of new virulence traits [9,16–18]. Asexual (clonal) reproduction of *Pca* occurs in oat, while a sexual reproduction phase is completed on an alternate host, *Rhamnus cathartica* (common buckthorn). Buckthorn is a native species to Europe and eastern Asia, which became invasive in North America after its introduction in the 1800’s [19]. Consequently, sexual recombination is thought to contribute to variation in the US population of *Pca*. In the US oat crown rust caused severe epidemics in the 1940s and 1950s and again in 1991 and 1993 where crop losses were very pronounced in the Northern region of the country [20]. Since then several outbreaks have occurred including the epidemic of 2014 when 8.7% of the US oat production was lost [16]. This period of increased disease prevalence coincides with a dramatic decline in oat production in the US from approximately 350 million bushels in 1990 to 90 million bushels in 2015 (75% reduction) [20,21], although economic and other factors are likely also contributors to this decline.

In North America, physiological races of *Pca* are defined using a set 40 oat differential lines containing different race-specific *R* genes (*Pc* genes), most of which are deployed in elite varieties [16,22]. The oat differential set is composed of common oat varieties that represent different *Pc* genes, which are numbered to reflect a chronological order of their description or release [23]. However, these are not a near-isogenic set and there has been limited genetic characterization of these lines [24–26], so it is unclear whether some of the lines contain multiple *Pc* genes or indeed if some *Pc* genes are duplicated in the set.

This study aims to examine genotypic changes of *Pca* isolates sampled from the US population before and after the most recent US oat crown rust epidemics (1991, 1993, 2014) to determine demographic and genetic factors that may have contributed to these outbreaks. Here, we have taken advantage of the annual surveys of oat crown rust conducted in the US to compare isolates of *Pca* collected in 1990 and in 2015. We show that there has been a dramatic increase in virulence of the US population between 1990 and 2015. Genome sequencing of these isolates detected evidence of changes in diversity, selection signatures, and presence/absence variation in predicted effector encoding genes and genome-wide association identified multiple putative *Avr* loci that may control virulence on various *Pc* resistance genes.

## Results

### Increased virulence of *Pca* sampled populations from 1990 to 2015

**Fig 1:**
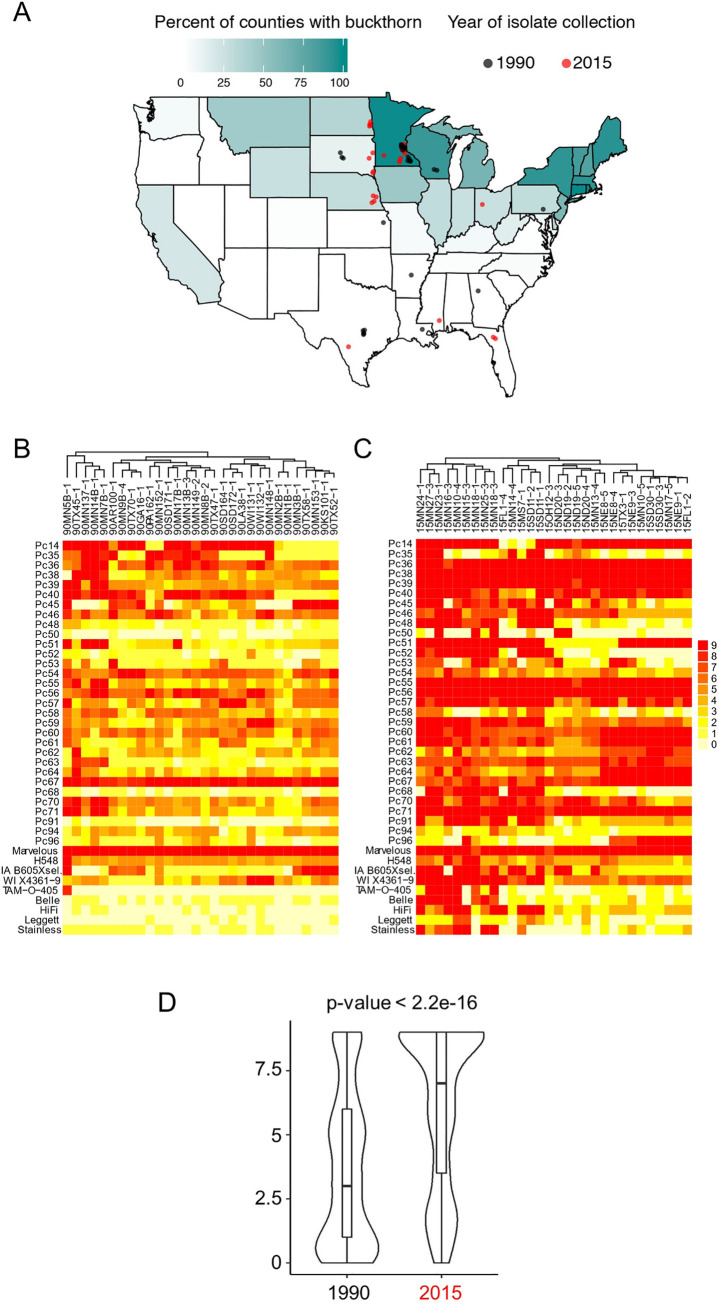
US oat production, distribution of *Rhamnus cathartica*, and virulence of *Puccinia coronata* f. sp. *avenae* populations. **(A)** Current distribution of *R*. *cathartica* (common buckthorn) in the US. The percentage of counties with recorded buckthorn in each state is indicated by the colored scale. Black and red dots in the map show collection locations for 1990 and 2015 populations, respectively. **(B)** Heatmap showing virulence profiles of isolates collected in 1990 on oat differential lines. **(C)** Heatmap showing virulence profiles of isolates collected in 2015 on oat differential lines. Infection scores were converted to a numeric scale (0 = resistance shown in yellow to 9 = susceptibility shown in red) for heatmap generation. Dendrograms reflect hierarchical clustering of columns and isolates with similar virulence patterns. (**D**) Distribution of infection scores of rust isolates (x-axis) by year (y-axis) against all differential oat lines. A box plot was drawn inside each violin plot to indicate median and inter-quartile range values. *p*-value was calculated using the Wilcoxon rank-sum test.

To assess changes in pathogen virulence and population structure between 1990 and 2015, we acquired 60 *Pca* isolates collected from across the US in 1990 and 2015 (30 individuals from each year, **Fig 1A and S1 Table**), representing timepoints prior and subsequent to the most recent epidemic years. These included samples from the Southern US where common buckthorn is absent, as well as Northern states where buckthorn is widely distributed (**Fig 1A).** The latter group also included isolates collected in the Minnesota Matt Moore buckthorn nursery, where oat varieties are grown adjacent to buckthorn hedges and sexual reproduction of *Pca* is prevalent. The infection type scores of all isolates were recorded after testing on the set of 40 oat differential lines (**Fig 1B and 1C)** including the universally susceptible oat variety Marvelous as a positive control for infection [16,23]. The overall virulence of the 2015 isolates (median = 7, interquartile range value = 5.5) was significantly higher than that of the 1990 isolates (median = 3, interquartile range value = 5) (**Fig 1D,** *p* < 2.2e-16 Wilcoxon rank sum test). There was a significant increase in virulence (*p*-value <0.05, Wilcoxon rank sum test) on thirty-six of the oat differential lines (excluding only *Pc14*, *Pc35*, *Pc40* and *Marvelous*) from 1990 to 2015 (**S2 Table**).

Ten oat lines (*Pc48*, *Pc50*, *Pc52*, *Pc68*, *Pc91*, *Pc96*, Belle, HiFi, Leggett, and Stainless) were immune to all of the 1990 isolates, but virulence to each of these lines was present in the 2015 population at frequencies of 10% to 35%, with the exception of *Pc94* for which only a single fully virulent isolate (3%) was detected in the 2015 population. A principal component analysis (PCA) using virulence scores showed a clear distinction between the 1990 and 2015 isolates (**S1A and S1B Fig)**. Within each year, there was little distinction between isolates based on geographic origin (**S1C–S1F Fig**). Overall, these results indicate that a dramatic increase in virulence occurred in the population of *Pca* during this 25-year period.

### Whole-genome sequencing reveals genetic differentiation of *Pca* sampled populations from 1990 and 2015

**Fig 2:**
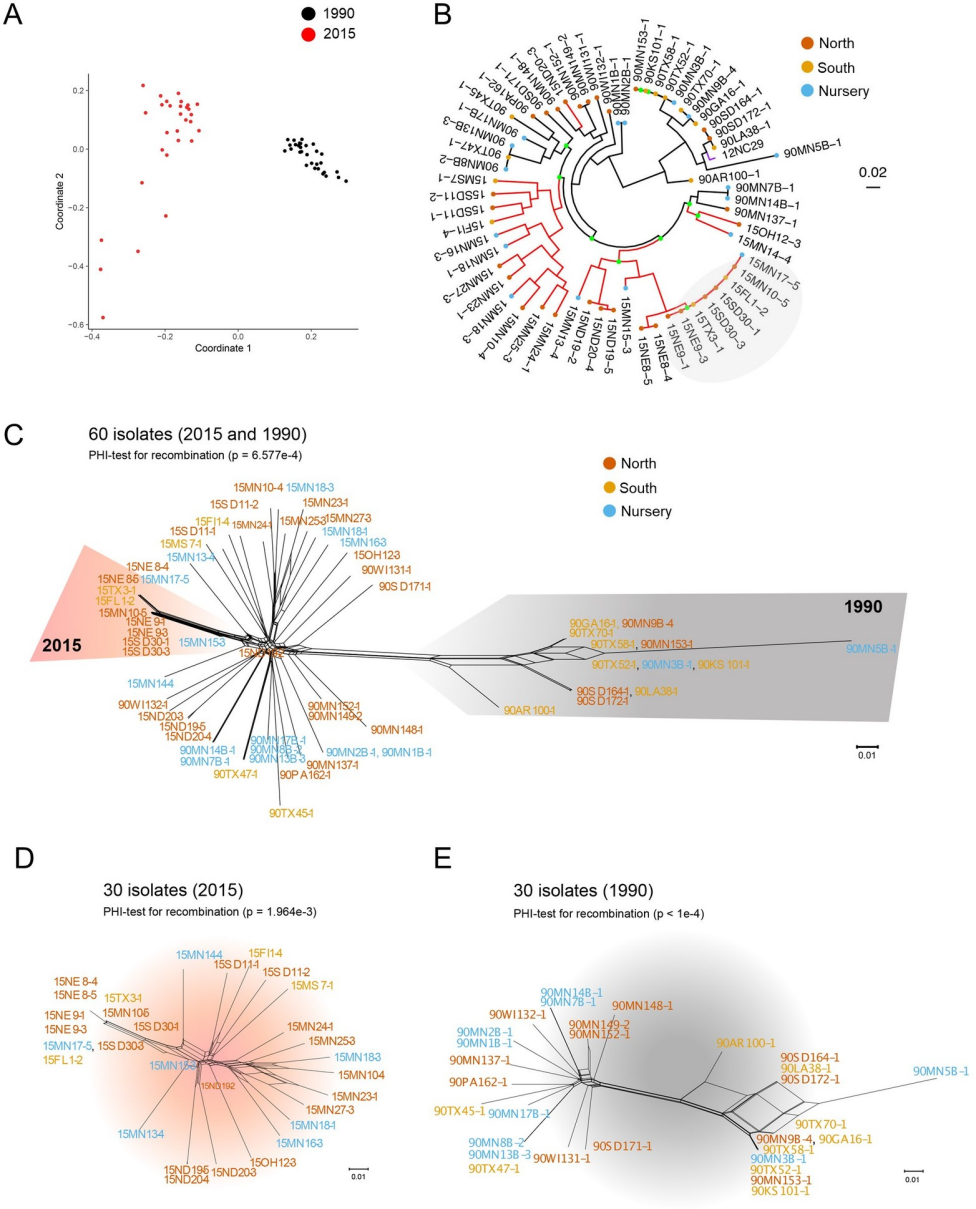
Population structure of *Puccinia coronata* f. sp. *avenae* in the US. **(A)** kWIP Genetic distance matrix of combined isolates from 1990 (black) and 2015 (red) collections. **(B)** Phylogenetic analysis using a maximum likelihood model with 2,582,690 variant sites and 500 bootstraps. The 1990 population is shown in black branches and the 2015 population is shown in red branches, with a purple branch for isolate 12NC29. Green dots on branches indicate bootstrap support of less than 50 percent, and colored dots on the tips of the tree reflect geographic origin if the isolates. Gray circle highlights an incipient clonal lineage. Scale bar indicates nucleotide substitutions per site. **(C)** Neighbor-net network of all *Pca* isolates based on 881,118 SNPs. Areas shaded in orange and grey indicate subpopulations belonging to 2015 and 1990 collections, respectively. PHI-score for recombination is also indicated. Color font in names of isolates reflect geographic origin. **(D)** Neighbor-net network of *Pca* isolates collected in 2015 based on 742,978 SNPs. **(E)** Neighbor-net network of *Pca* isolates collected in 1990 based on 912,086 SNPs.

We sequenced gDNA from each isolate (*n* = 30 individuals per collection year) with Illumina 125 bp paired-end reads (**S1 Table**) to an average coverage of 81X (54 -108X) of the primary contigs of the 12SD80 reference genome (i.e., haploid genome content) [22] (**S2 Fig and S1 Table**). For genome-wide analysis of sequence variation we used FreeBayes [27] to call Single Nucleotide Polymorphisms (SNPs) from reads mapped to the 12SD80 primary contig reference genome [28]. Read allele frequencies at heterozygous positions in each isolate showed the normal distribution [29] expected for a single pure genotype and no mixed genotype samples were detected (**S3 Fig**). A kWIP *k*-mer similarity analysis on the raw sequence data [30] separated the isolates from 1990 and 2015 (**Fig 2A**), indicating genetic divergence between these temporally separated populations. This separation was independent of geographic origin of the isolates (Southern states, Northern states or buckthorn nursery; **S4A Fig**), suggesting that the US *Pca* population has evolved as a whole over this period, rather than local populations evolving independently. No clustering was found based on geographic origin of the isolates within either population (**S4B and S4C Fig**), suggesting substantial regional migration. Similarly, a discriminant analysis of principal components (DAPC) [31,32] clearly separated the 2015 population from the prior 1990 population (**S5A Fig**), but found limited separation between isolates based on geographic regions within each year (**S5B Fig**).

### Sexual and asexual processes have contributed to *Pca* population diversity

*Pca* populations in the US can display a mixed reproductive system as they can undergo multiple cycles of asexual (clonal) reproduction within a single cropping season while a single round of sexual reproduction can occur between seasons if buckthorn is present [16]. Asexual clones can also be maintained across seasons. To assess evolutionary relationships between isolates and explore the extent of clonality, we first generated a maximum-likelihood (ML) phylogenetic tree using all biallelic SNPs (2,582,690). In this analysis most individuals were separated by deep branches, but several very closely related individuals grouped into clades that likely represent clonal lineages that were prevalent in each year (**Fig 2B**). In some cases clonal isolates were collected from distant states indicating that they were geographically widely dispersed (i.e., isolates from MN, TX and KS for the 1990 population and MN, FL, SD, TX and NE for the 2015 population, **Fig 2B**). This is consistent with the known long-distance dispersal of rust fungi and indicates substantial migration within the large geographical region.

The deep branches separating most isolates in the phylogenetic tree are consistent with the contribution of genetic recombination to the generation of unique individuals. Furthermore, the number of population clusters (Kmax) identified using the Bayesian Information Criterion (BIC) was *k* = 13 and *k* = 12 for the 1990 and 2015 isolates, respectively, which is close to separating the populations into single individuals or clones. To better represent relationships within a potentially recombining population, we generated a neighbor-net network (SplitsTree) from SNP data for samples from both collection years **(Fig 2C)**. The extensive reticulation in this network indicates frequent past recombination events, which was supported by PHI-test scores [33]. The neighbor-net network did not support segregation of isolates by geographic region or year. Interestingly, we detected one branch with long parallel reticulated paths in the 2015 population that contains the same eight isolates highlighted in the ML phylogenetic tree, which may represent an incipient clonal expansion. Neighbor-net networks generated separately for each sampling year also provided evidence for recombination, including for isolates that derived from geographic regions without the sexual host for *Pca* **(Fig 2D and 2E)**. Isolates from the same geographic regions were dispersed in the networks, again indicating a lack of geographic substructure in these populations and consistent with the wide dispersal of isolates.

The *F*_ST_ fixation index [34] between the 1990 and 2015 populations (mean *F*_ST_ = 0.039) (**S6A Fig**) is consistent with a strong influence of sexuality on the *Pca* populations. *F*_ST_ values between isolates from different geographical regions were low in 1990, while in 2015 all pairwise *F*_ST_ values between regions were negative, again supporting a lack of geographical differentiation within the populations (**S6B Fig**). We also calculated the standardized index of association (*r_d_*) [35] as an estimate of genome-wide linkage disequilibrium (LD). The *r_d_* distributions for each population calculated using 100 sets of 10,000 random SNPs were between the values observed for simulated datasets with 75% and 100% LD [36] (**S6C Fig**). This suggests that, despite the occurrence of substantial sexual recombination, the evolutionary history of the *Pca* populations is also influenced by asexual reproduction and the persistence of clones, as detected in the phylogenetic analysis (**Fig 2B**). The *r_d_* distribution of the 2015 population was lower (Kruskal-Wallis test, *p* < 0.05, *X*^2^ = 577.07) and in a different rank than that of 1990, possibly due to a higher extent of clonal reproduction or different demographic scenarios.

### Identification of *Avr* loci by genome-wide association

**Fig 3:**
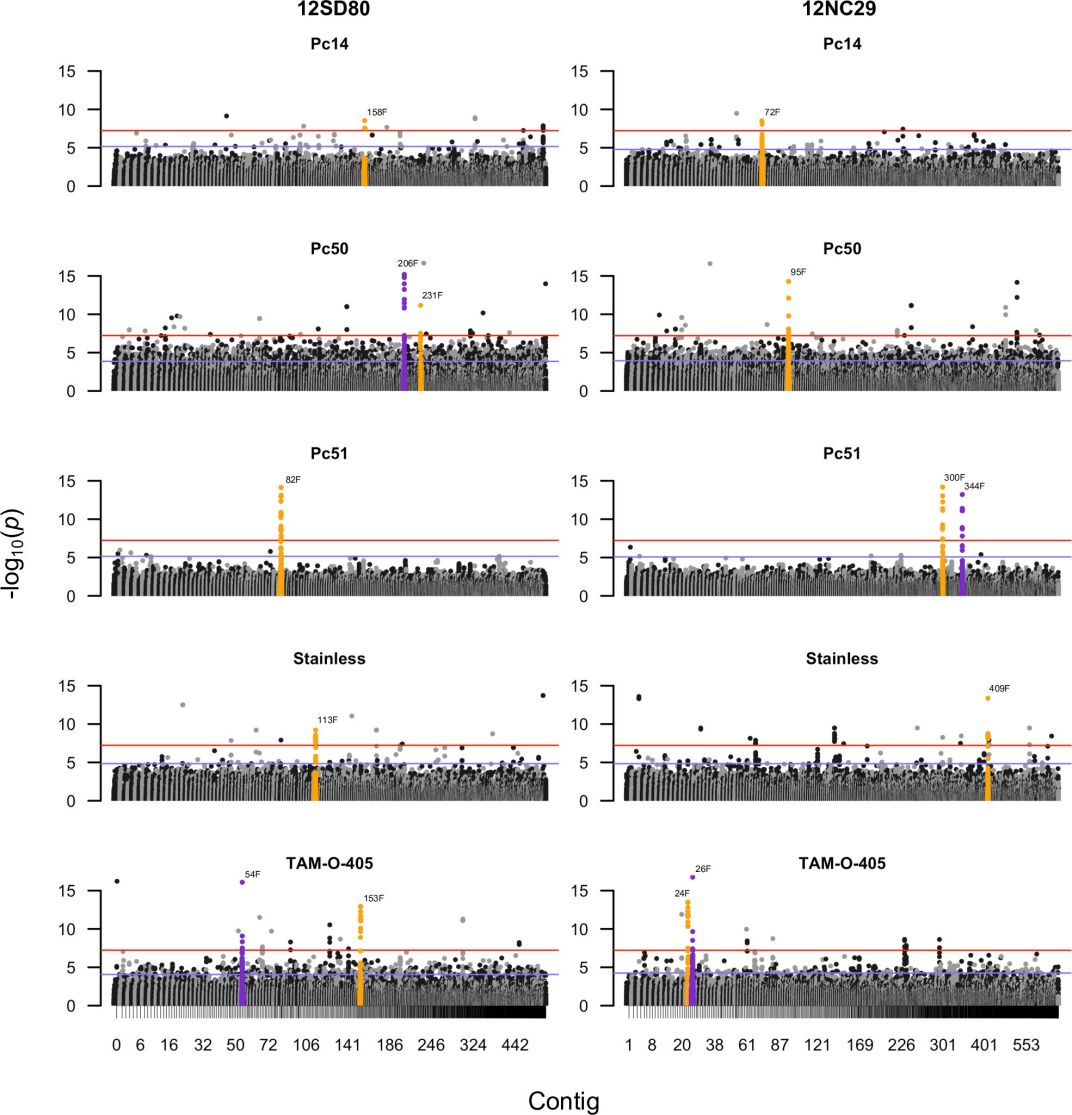
GWAS for virulence of *Pca* towards oat differential lines. Manhattan plots showing SNP association values for virulence to oat lines *Pc14*, *Pc50*, *Pc51*, Stainless and TAM-0-405 across primary contigs in the 12SD80 and 12NC29 genome assemblies. Highlighted SNPs in orange are derived from contigs with significant association peaks and containing predicted effector genes, whereas SNPs in purple are derived from significant contigs without any predicted effector genes. Red and blue horizontal lines denote Bonferroni significance threshold (α = 0.05/total number of markers) and 5% false discovery rate threshold, respectively.

**Fig 4:**
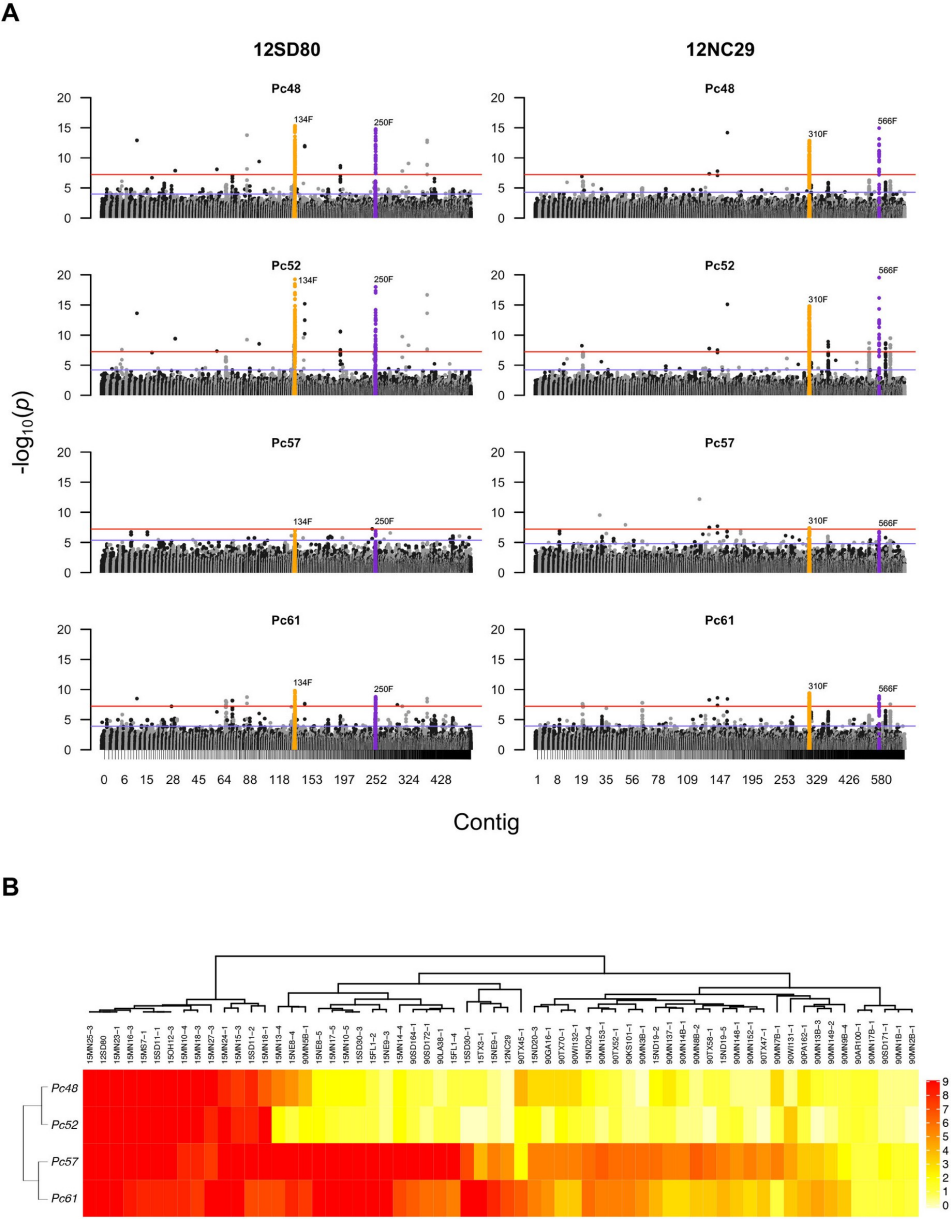
GWAS for virulence of *Pca* towards oat differential lines *Pc48*, *Pc52*, *Pc57*, and *Pc61*. **(A)** Manhattan plots showing SNP association values for virulence to *Pc48*, *Pc52*, *Pc57*, and *Pc61* across primary contigs in the 12SD80 (contig 134F and 250F) and 12NC29 (contigs 310F and 566F) genome assemblies. Highlighted SNPs in orange indicate contigs with significant association peaks and encoding predicted effector genes, whereas SNPs in purple represent significant contigs without any predicted effector genes. Red and blue horizontal lines denote Bonferroni significance threshold (α = 0.05/total number of markers) and 5% false discovery rate threshold, respectively. **(B)** Heatmap showing virulence profiles of all isolates collected including 12SD80 and 12NC29 on *Pc48*, *Pc52*, *Pc57*, and *Pc61*. Infection scores were converted to a numeric scale (0 = resistance shown in yellow to 9 = susceptibility shown in red) for heatmap generation.

To identify genomic locations that may be linked to virulence on each of the 40 differential lines we performed a genome wide association study (GWAS) using whole genome SNP data of all 62 *Pca* isolates, including the two reference isolates 12SD80 and 12NC29 [22]. Evaluation of Manhattan plots produced in two independent analyses using either the 12SD80 or 12NC29 primary contig assemblies as the reference detected several regions showing association with virulence on fifteen of the differential lines **(Figs 3 and 4 and S7–S10)**. Importantly, the two analyses on the different genome references were in agreement and identified association peaks for the same set of virulence phenotypes in syntenic regions between the two genomes (**S11 Fig**). We examined gene content in contig regions with multiple significantly associated SNPs (**Table 1**). For four differential lines (*Pc14*, *Pc50*, *Pc51* and Stainless) we found a single peak of highly significant association in a *Pca* genomic region that was unique to that differential line (**Fig 3**). This is consistent with each of these lines containing a single resistance gene that recognizes a unique single *Avr* locus. In each case the genomic regions identified in 12NC29 and 12SD80 were syntenic (**S11 Fig**). In each of these cases the associated region contained a single predicted secreted effector gene which represents a strong candidate for the corresponding avirulence genes *AvrPc14*, *AvrPc50*, *AvrPc51* and *AvrPcStainless* (**Table 2**). For the differential line TAM-O-405, we detected significant association peaks on two contigs in each of the 12SD80 and 12NC29 assemblies, which may indicate that these contigs are adjacent in the genome and represent a single associated region. Six effector candidates were detected within the associated regions which could represent the *AvrTAM-O-405* gene. In each case, sequence differences occurred between the alleles of the *Avr* gene candidates present in 12NC29 and 12SD80 (**Table 2**), consistent with their different virulence phenotypes (12NC29 is avirulent and 12SD80 virulent) on all these *Pc* genes [22]. For instance, the *AvrPcStainless* allele in 12SD80 contains a 1 bp deletion causing a frameshift, while other variants include amino acid changes.

**Table 1:** Genomic regions and gene content in 12NC29 and 12SD80 identified through a genome-wide association of virulence to specific *R* genes.

Genome reference	Contig name	Contig size (bp)	Significant (sig.) region coordinates (Bonferroni threshold)	Predicted genes in sig. region	Predicted effectors in sig. region (secreted)
12NC29	9F	672008	193626:195366	0	0
	24F	461715	124539:195618	25	6
	26F	478440	314446:378362	13	0
	32F	447225	329482:350648	1	0
	72F	312302	56042:96556	11	1
	95F	278016	137388:193439	5	0
	268F	131982	25861:112196	19	2
	300F	121826	1532:49164	4	0
	310F	118716	67:65083	11	2
	344F	104484	959:8967	1	0
	359F	110554	12363:107918	10	1
	409F	90341	45605:81998	1	1
	566F	55459	207:36028	7	0
12SD80	8F	781985	468242:472082	0	0
	54F	345470	113617:158789	12	0
	82F	319979	184530:259788	12	1
	113F	291735	234171:289309	7	0
	134F	246564	163696:224784	9	0
	153F	234317	205949:230879	6	2
	158F	213051	105906:180622	9	1
	174F	209206	3866:124378	21	0
	206F	179499	143959:167074	2	0
	231F	159934	10738:33210	2	1
	250F	152435	10947:130512	19	0
	301F	125094	93945:102540	3	1
	361F	94012	89856:93149	0	0
	484F	58973	263:58653	13	2

**Table 2:** Summary of candidate *Avr* genes detected by genome-wide association of virulence to specific *R* genes.

Pc* gene in oat differential lines	12NC29 fungal genome reference		12SD80 fungal genome reference	
	Gene ID	Genomic location	Gene ID	Genomic location
*Pc14*	PCA_NC_11327	72F, 61808:62443	Not annotated, 12-bp deletion	158F, 184315:184961
*Pc50*	Not annotated, 24-bp deletion	95F, 183642:185716	PCA_SD_14394	231F, 24395:26467
*Pc51*	PCA_NC_18844	300F, 29455:31259	PCA_SD_06399	82F, 208451:210343
*Stainless*	PCA_NC_25211	409F, 72346:73101	Not annotated, 1-bp deletion	113F, 264085:264839
*TAM-O-405*	PCA_NC_02201	24F, 130946:138677	PCA_SD_11290	153F, 212358:220090
	PCA_NC_02203	24F, 144882:146281	PCA_SD_11292	153F, 226548:227693
	PCA_NC_02204	24F, 149581:150941	Not annotated, 99% DNA identity	153F, 230993:232353
	PCA_NC_02218	24F, 180685:181004	Not annotated, 99% DNA identity	301F, 108556:108875
	PCA_NC_02219	24F, 181344:182891	PCA_SD_12454	301F, 105881:108216
	PCA_NC_02221	24F, 191069: 191714	PCA_SD_12452	301F, 97847: 98492
*Pc48*/*52*/*57*/*61*	PCA_NC_21901	310F, 37148:37583	Not annotated, 8-bp deletion	134F, 198963:199390
	PCA_NC_21903	310F, 49014:49773	Not annotated, 300-bp deletion	134F, 172222:172515, 134F, 211130:211471
*Pc38*/*39*/*55*/*63*/*70*/*71*	PCA_NC_25789	359F, 85929:87053	Not annotated, 99% DNA identity	484F, 13219:14343
	Not annotated, 99% DNA identity	359F, 107339:108907	PCA_SD_22939	484F, 6620:8188
	Not annotated, 3-bp deletion	359F, 94186:95497	PCA_SD_22940	484F, 10166:11478
	PCA_NC_15079	268F, 41069:43302	Not annotated, 6-bp deletion	174F, 96514:98741
	PCA_NC_15084	268F, 60920:62265	PCA_SD_13927	174F, 77571:78916

*The gene nomenclature for race-specific resistance to crown rust disease stands for *Puccinia coronata (Pc)*

The remaining ten differential lines fell into two groups of four and six, with members of each group all detecting significant associations with the same genomic region in *Pca*. One group included *Pc48*, *Pc52*, *Pc57* and *Pc61*, which detected virulence associations with syntenic genomic regions in 12NC29 (contig 310F and 566F) and 12SD80 (contig 134F and 250F) (**Figs 4A and S9**). In 12NC29 these regions include two genes (PCA_NC_21901 and PCA_NC_21903 in contig 310F) encoding predicted secreted proteins which represent *Avr* effector candidates (**Table 2 and S12 Fig**). Orthologous sequences of these genes were identified in contig 134F of 12SD80; however, these sequences include 8 bp and 300 bp deletions, respectively, and were not annotated. These deletions disrupt the coding sequences of the two genes, which is consistent with the virulent phenotype of 12SD80. Comparison of the virulence phenotypes of all 62 isolates to these four resistance genes (**Fig 4B)** showed that the resistance spectrum of *Pc48* and *Pc52* to these isolates was very similar, indicating that they may either contain the same gene conferring the resistance phenotype, or different genes/alleles that recognize the same Avr effector. Likewise, *Pc57* and *Pc61* showed very similar resistance spectra and may contain the same gene. However, the resistance profiles of *Pc48*/*52* and *Pc57*/*61* differed from each other but showed some overlap in that isolates virulent on *Pc48*/*52* were also virulent on *Pc57/61*, and isolates avirulent on *Pc57* and *Pc61* were also avirulent on *Pc48*/*52*. Nevertheless, many isolates avirulent on *Pc48*/*52* were virulent on *Pc57*/*61*. The association signal for *Pc57*/*61* with this genomic region was weaker than for *Pc48*/*52* suggesting that this locus may only contribute partially to virulence on *Pc57/61*.

Virulence phenotypes to the six resistance gene differentials *Pc38*, *Pc39*, *Pc55*, *Pc63*, *Pc70* and *Pc71* all showed association peaks on four contigs in 12SD80 (contigs 8F, 174F, 484F, 361F) and four in 12NC29 (Contigs 9F, 32F, 268F, 359F) (**S7 and S13 Figs**). 12NC29 is avirulent for each of these resistance genes while 12SD80 is virulent. Contigs 268F and 359F in 12NC29 are syntenic to 174F and 484F in 12SD80 respectively (**S11 Fig**) [22] and contain five predicted effector genes (**Tables 1 and 2**). The gene PCA_NC_25789 occurs in contig 359F of 12NC29 and a 99% identical sequence was identified in contig 484F of 12SD80 but had not been annotated. Two other genes annotated in 12SD80 contig 484F (PCA_SD_22939 and PCA_SD_22940) contained homologous sequences in 12NC29 contig 359F that were not annotated. A homologous sequence in contig 174F of 12SD80 was found for PCA_NC_15079 in contig 268F of 12NC29. This sequenced was not annotated as a gene likely due to a 6 bp-deletion. We also identified that for gene PCA_NC_15084, which is also present in contig 268F of 12NC29 there was a homologous gene PCA_SD_13927 in contig 174F, which was not annotated to encode a secreted protein.

Identical resistance phenotypic profiles were found for *Pc63* and *Pc38*, suggesting that these differential lines may contain the same resistance gene (**S13 Fig**). *Pc71*, *Pc55*, and *Pc39* also showed very similar profiles to each other, suggesting that these lines contain the same or related resistance genes. However, two *Pca* isolates distinguished *Pc38*/*63* from *Pc39*/*55*/*71*: one was avirulent on *Pc38*/*63* and virulent on *Pc39*/*55*/*71* while the second showed the opposite response. Such contrasting phenotypic patterns clearly distinguished these two groups as showing different recognition specificities. Again, this could indicate that the resistance genes are allelic and recognize different versions of the same *Avr* gene, or that they recognize two closely linked *Avr* genes in the same genomic region. *Pc70* showed a similar resistance profile to *Pc39*/*55*/*71* but was resistant to additional isolates, indicating that it may contain the same resistance gene in combination with an additional gene of a different specificity. This is consistent with the weaker association observed between this region and *Pc70* virulence.

### Evidence for selective sweeps in the *Pca* genome

To detect signatures of selection in genes relevant to pathogenicity, we calculated nucleotide diversity (*π*), Watterson’s *θ* and Tajima’s *D* for genes encoding putative effectors (*n* = 529) and all other predicted genes (*n* = 16,719) [22]. Diversity was slightly higher in predicted effectors than other genes within the 1990 population but there was no difference between these groups within the 2015 population (**S14A and S14B Fig and S3 Table**). Tajima’s *D* for effector genes was significantly lower than for the remainder of genes in both years (*p* < 0.05 Wilcoxon rank sum test, **S14 Fig and S3 Table**). Consistent with this, the lowest quartile of the distribution of Tajima’s *D* values was lower for effectors than for all genes. This greater proportion of lower negative values reflects an excess of rare alleles in some effector genes, consistent with the genes being more frequent targets of directional selection and selective sweeps.

**Fig 5:**
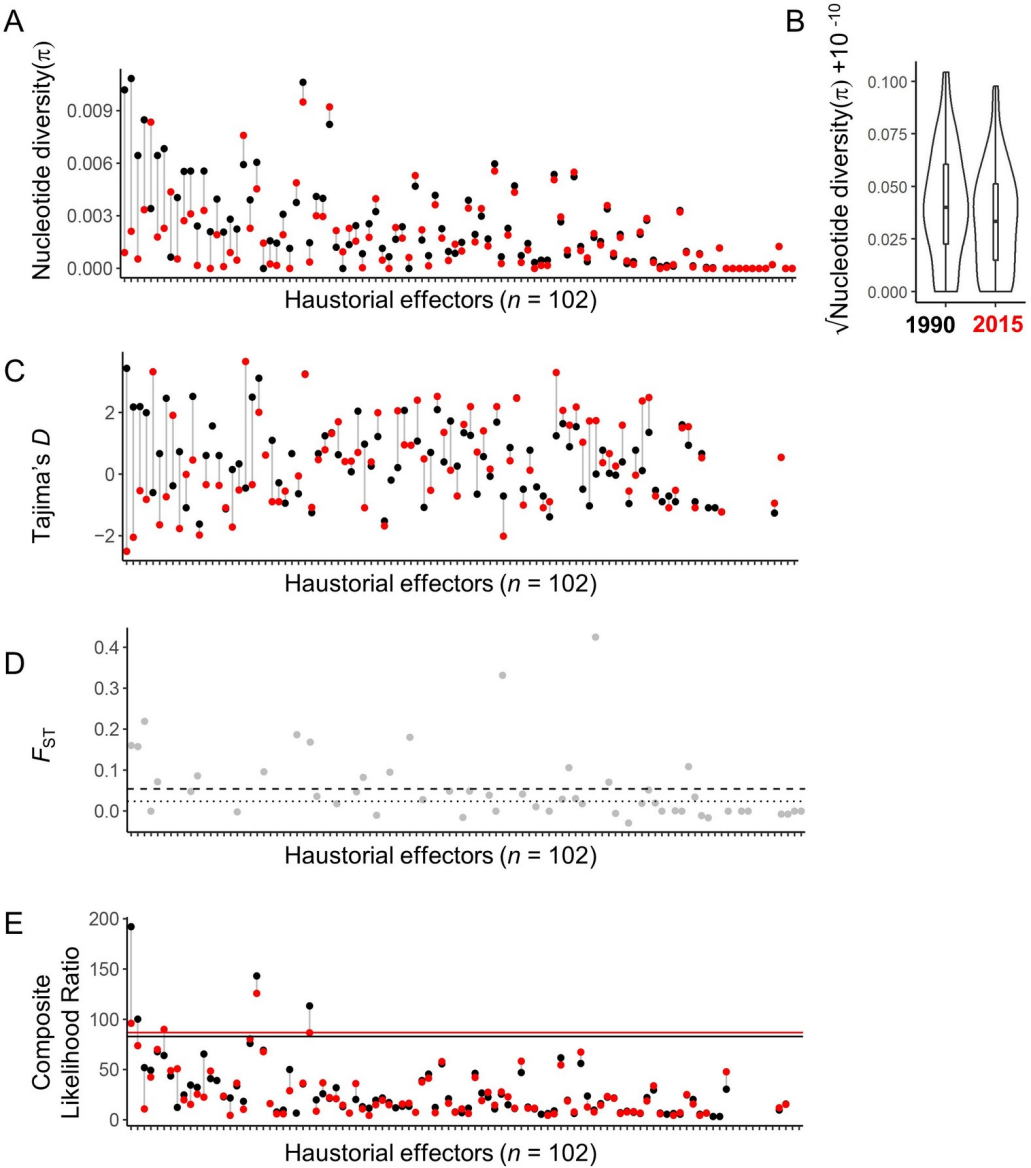
Evidence for selection in haustorially-expressed predicted effector repertoires. **(A)** Nucleotide diversity (π) values for predicted effectors in 12SD80 with haustorial expression (*n* = 102, x-axis) using 1990 (black) and 2015 (red) datasets. **(B)** Distribution of nucleotide diversity values (y-axis) for predicted effectors in 12SD80 with haustorial expression in 1990 and 2015 (x-axis). A box plot was drawn inside each violin plot to indicate median and inter-quartile range values. **(C)** Tajima’s *D* values for predicted effectors in 12SD80. **(D)** *F*_ST_ values for predicted effectors in 12SD80 when comparing 1990 and 2015 datasets. The mean for all genes (0.05) is shown by a dashed line at and whereas the dotted line represents the median value (0.024). **(E)** Composite likelihood ratio (CLR) scores for predicted effectors of 12SD80. The horizontal lines in **E** show the 95% percentile thresholds for 1990 (black) and 2015 (red). List of haustorial effectors and raw data is provided in table S4.

Next, we closely examined a subset of effector candidates with high expression in haustoria (*n* = 102) [22] to detect those showing a decrease in population-wide genetic diversity and Tajima’s *D* since 1990, a hallmark of a selective sweep. Several haustorial effectors show a decrease in *π* since 1990 (**Fig 5A** and **S4 Table**), consistent with the genome wide reduction of diversity over this time period (**S14E Fig**). The overall distribution of *π* values for haustorial effectors in 2015 was not different to that in 1990 (*p* = 0.06, Wilcoxon rank sum test) (**Fig 5B)**. However, the four predicted effectors with the largest decrease in *π* between years (PCA_SD_03307, PCA_SD_01254, PCA_SD_15364, PCA_SD_06058) also exhibited a strong reduction in Tajima’s *D* resulting in negative values (**Fig 5C** and **S4 Table**), consistent with a selective sweep. Three of these also showed relatively high *F*_ST_ values (**Fig 5D**), consistent with selection of distinct alleles in the 1990 and 2015 populations. A few other effectors also showed high *F*_ST_ values (**S4 Table**). As a further test for selective sweeps in the haustorial effector gene set, we also calculated the composite likelihood ratio (CLR) [37], which uses variation in the site-frequency spectrum along the genome (**Fig 5E** and **S4 Table**). The effector gene with the largest decrease in nucleotide diversity (PCA_SD_03307, first effector on *x*-axis of **Fig 5A**) also showed a high CLR value (above the 95th percentile for each year), supporting a recent selective sweep. A BLASTp search with the PCA_SD_03307 protein sequence identified homologous genes in the wheat stem rust fungus and stripe rust fungus, which are also reported to have haustoria and infection-specific expression (**S5 Table**).

**Fig 6:**
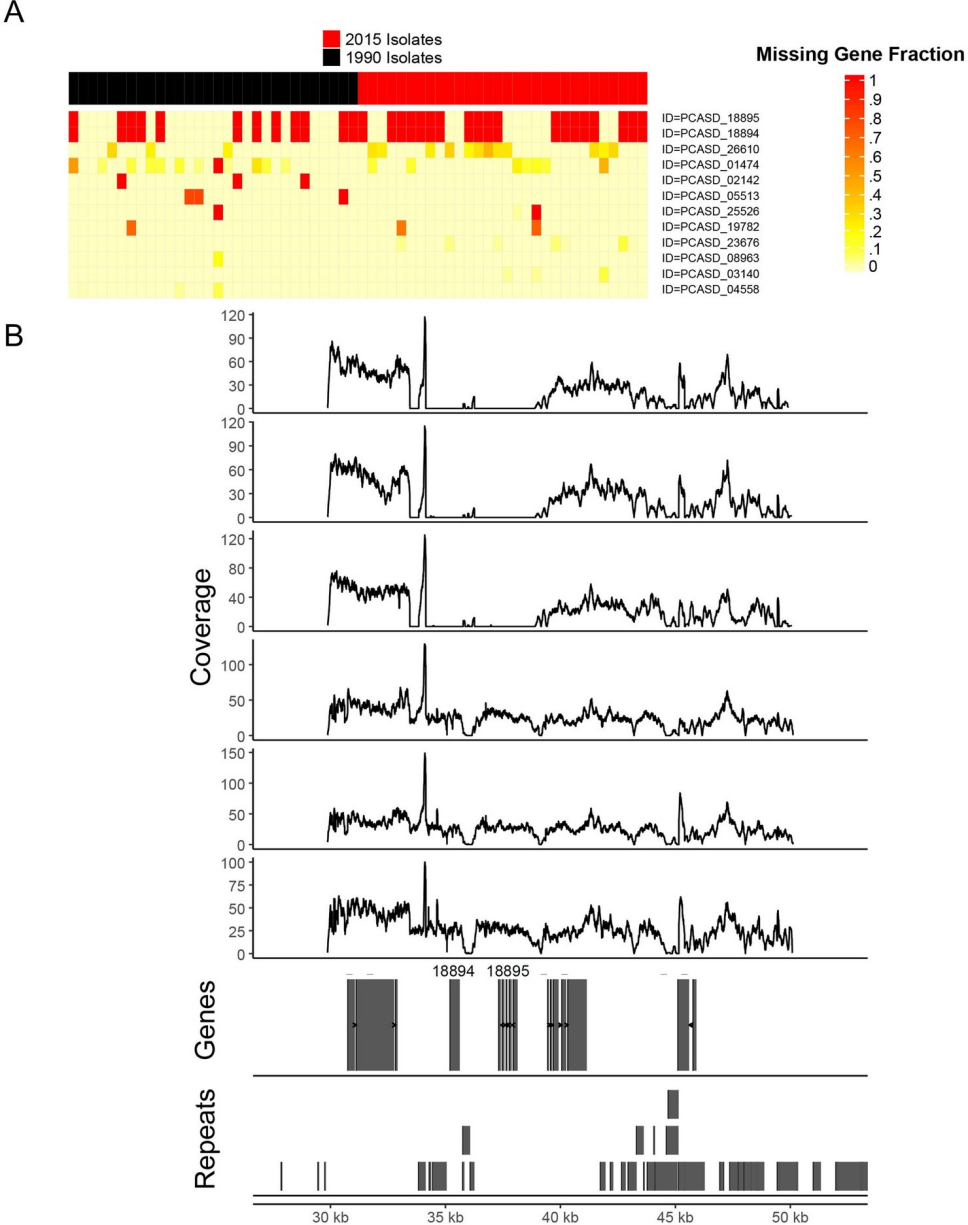
Detection of gene deletions in haustorially-expressed predicted effector repertoires in 1990 and 2015. **(A)** Presence/absence variation in 12 haustorially-expressed predicted effectors in 1990 and 2015. All isolates are represented as a black (1990) and red (2015) top bar (x-axis) above missing gene fraction matrix (y-axis). The heatmap indicates the missing fraction of each gene model as assessed with read mapping coverage. Color scale indicates 0 = 0% deletion to 1 = 100% deletion. **(B)** A detailed view of read coverage across the genomic region surrounding PCASD_18894/18895. The top six tracks show mapping coverage in the surrounding region from 3 representative isolates with (top) and without (bottom) the deletion. The bottom two tracks show gene and repeat locations, respectively.

Twelve haustorial effectors showed presence/absence polymorphisms (**Fig 6A and S5 Table**), based on per-base read coverage data. PCA_SD_18894 and PCA_SD_18895 were notable in that they both showed complete deletion in 31 isolates (12 and 19 from 1990 and 2015, respectively). These two effectors are adjacent in the genome, and close examination of read mapping across this region containing these genes showed a single deletion of ~5 kb spanning both genes in some isolates (**Fig 6B**). The region surrounding these genes is repeat-rich, suggesting that transposon activity could have facilitated the deletion. The protein sequence of PCA_SD_18894, had no homology to any proteins in the NCBI BLASTp database, while PCA_SD_18895 had homology to predicted effectors expressed in haustoria in the wheat stripe and stem rust fungi (E-Values all < 1e-30, average percent identity 35%, **S5 Table**). We used a Canonical Correspondence Analysis (CCA) to explore the relationship between virulence phenotypes on the oat differential set and presence/absence variation (PAV) of effectors (**S15 Fig**). Isolate phenotypes were not strongly grouped by effector PAV, with many differential line phenotypes clustering at the origin of the PAV vectors. However, *Pc96* is positioned far along the PCA_SD_26610 vector, suggesting some contribution of PAV in this effector to the virulence phenotype.

To identify genomic regions that may have been under selection, nucleotide diversity (*π*), Watterson’s *θ* and Tajima’s *D* were calculated for each contig in the genome assembly (**S14D–S14F Fig**). The distributions of both *π* and Watterson’s *θ* values were significantly higher for the 1990 population than for the 2015 population (mean *π* in 1990 and 2015 = 0.0026 and 0.0022, mean Watterson’s *θ* = 0.0019 and 0.0016, respectively, *p* < 10^−7^ Wilcoxon rank sum test) suggesting that the US continental population of *Pca* was more diverse in the past. Both populations displayed positive Tajima's *D* mean values in each year reflecting an excess of alleles of intermediate frequencies. Tajima’s *D* values were significantly higher in 2015 than in 1990 (1.29 versus 1.03, respectively, *p* < 10^−7^ Wilcoxon rank sum test) (**S14F Fig**), consistent with a decrease in population size due to the decline in oat acreage over this time. Substantial negative Tajima's *D* mean values (< -1.0) were detected in 14 contigs (total of 1.5 Mbp) in the 1990 population and in 13 contigs (1.4 Mbp) in the 2015 population (**S6 Table**), which may indicate regions of the genome that had been subject to recent selective sweeps. Given the relatively small size of most of these contigs, it is possible that they represent only one or a few contiguous genomic regions. Notably, contig 484F containing the putative *AvrPc38/39* locus returned a Tajima’s *D* value of -1.98 in the genetic diversity analysis in 2015, but was 3.22 in 1990 (**S6 Table**) suggesting a selective sweep on this region occurred between these two time points. This is consistent with the observation that this locus has swept to virulence in the entire 2015 sampled population, compared to the segregating virulent and avirulent phenotypes in the 1990 population (**Fig 1B and 1C**).

## Discussion

Understanding how pathogen populations evolve in response to the deployment of resistance genes in crops is important to minimize the risk of disease outbreaks. In the past 30 years the genetic resistance present in most oat varieties has been overcome in the US, as well as other parts of the world [16]. Here, we compare *Pca* isolates collected from across oat growing regions of the US in 1990 and 2015. This comparison documents the dramatic shift in the virulence profile of *Pca* populations in the US towards an increase in the frequency of virulent isolates on most *Pc* genes over this time period. Genome-wide sequence analysis indicates that this virulence increase is associated with a significant genetic change in the *Pca* population over this time and provided some insights into the underlying demographic processes and locus-specific effects that have contributed to this change. While 2015 population is clearly distinguished from the 1990 population, the low *F*_ST_ value between these populations suggests that the latter is largely derived from the former, rather than representing a replacement population derived from migration of an exotic broadly virulent *Pca* isolate. Thus, this dramatic virulence shift may be explained by the *in situ* evolution of the pathogen population within the North American continent, although some contribution from gene flow cannot be excluded. The lower genetic diversity and higher Tajima’s *D* in the 2015 population is consistent with the effects of a recent population bottleneck. The drastic decline in oat acreage in the US over this period [21,38,39] and the selection of particular virulence gene combinations in response to cultivation of resistant oat varieties may be factors contributing to this decrease in pathogen diversity. Analysis of additional pathogen collections from the intervening years, especially the epidemic years of 1991 and 1993, could provide further information about when these changes to the *Pca* population occurred and if it resulted from a major event or gradual change over this time.

The alternate host of *Pca*, *R*. *cathartica*, is widespread in the Northern US [40], but it was not known to what extent sexual reproduction contributes to the population dynamics and evolution trajectory of *Pca*. We find evidence that genetic recombination as demonstrated by the reticulation of the phylogenetic neighbor-net, contributes strongly to variation within populations, but that clonality and migration are also important factors. *F*_ST_ values observed for these populations are similar to those observed in sexual populations of *M*. *larici-populina*, and substantially lower than those observed between asexual populations [41,42], which supports the contribution of sexuality to the population structure. However, LD simulations were also consistent with the influence of asexual reproduction (clonality) and phylogenetic analysis detected a few clonal lineages within each year. The role of both sexual and asexual reproduction in the evolutionary history of these *Pca* populations is not surprising as the pathogen can complete one sexual cycle per year, whereas multiple cycles of clonal reproduction (potentially every two weeks) occur during the crop growing season [43]. The combination of asexual and sexual cycles in plus the high degree of gene flow in the US population likely allows rapid responses to selective pressures. Another process that could contribute to genetic diversity is nuclear exchange by somatic hybrization, which was observed to give rise to new genotypes in the related species *P*. *graminis* f. sp. *tritici* [15]. The reservoir of wild oats (i.e., *Avena strigosa*) that occur on highway verges and in proximity to commercial oat fields [44,45] can also serve as an extended green bridge that can allow the persistence of clonal lineages of the pathogen between seasons. Further comparative analysis including large collections from *Pca* isolates derived from *R*. *cathartica* will be key to fully examine the contribution of sexuality to the genetic diversity of *Pca* populations. Future comparisons with *Pca* populations from geographically isolated regions (i.e., other continents) where common buckthorn has not been introduced will help to elucidate the role of clonality and sexuality in evolution of virulence in *Pca*.

We observed clear evidence of high migration of *Pca* within the continental US, particularly through the isolation of certain clonal lineages from widely distributed sites across the sampling area. This is consistent with the airborne nature of *Pca* urediniospores, and the postulated migration pathway (the Puccinia pathway) that contributes to gene flow [46]. This suggests that the entire US represents a single metapopulation characterized by substantial migration and gene flow between regions. However, the degree of geographical structure within the populations was difficult to measure in this study due to relatively low numbers of samples corresponding to different regions. We did not detect strong evidence for structure within the 1990 or 2015 populations based on kWIP or DAPC analyses and observed some admixture between geographical regions. However, the sample size available from these years was limited and future collections of larger samples from across the country will be necessary to further clarify the extent to which local populations may be differentiated and over what time periods this could be maintained.

Genome-wide association studies (GWAS) in various pathogens have shown their utility for effector discovery (42). These approaches have been underutilized in rust fungi. This is partly due to the highly clonal nature of many rust populations, such as the wheat stripe rust [47] and stem rust fungi [15,41], where only a few clonal linages dominate global populations. The high diversity and sexual recombination in the US *Pca* populations allowed us to use a genome-wide association approach to discover candidate *Avr* genes corresponding to multiple race-specific *Pc* resistance genes. We found genomic regions showing significant associations with virulence phenotypes on fifteen different *Pc* genes. In four cases (*Pc14*, *Pc50*, *Pc51*, *Stainless*), we identified a single effector candidate within the genomic regions showing an association with the avirulence phenotype. In each case the allele present in the virulent 12SD80 isolate was different to that in avirulent 12NC29, consistent with their different infection phenotypes. Thus, PCANC_11327, PCANC_10462, PCANC_18844 and PCANC_25211 are strong candidates for the avirulence genes *AvrPc14*, *AvrPc50*, *AvrPc51* and *AvrPcStainless* respectively. In the case of *TAM-O-405* the genomic region associated with virulence in 12NC29 and 12SD80 contained five effector genes which are candidates for *AvrPcTAM-O-405*.

Interestingly, we identified the same genomic region associated with virulence to four *Pc* differential lines containing the resistance genes *Pc48*, *Pc52*, *Pc57*, and *Pc61*. The similarity in resistance profiles of *Pc48* and *Pc52* suggests that these differential lines may contain the same resistance gene or closely related alleles. Likewise, the *Pc57* and *Pc61* differentials also show similar resistance profiles and may represent a duplication of the same resistance gene (or closely related alleles) in the differential set. *Pc57*/*61* show resistance to a subset of isolates that are avirulent to *Pc48*/*52*, which is consistent with a scenario in which the product of these genes recognize the same effector, but a separate virulence factor (inhibitor locus) in the rust fungus suppresses recognition by *Pc57*/*61*. Similar situations have been described in flax rust and in wheat powdery mildew [48,49]. Alternatively, *Pc57*/*61* and *Pc48*/*52* may represent variants of the same resistance gene that differ in the recognition spectrum for the same corresponding Avr protein variants. This was observed for some *Pm3* alleles in wheat which recognize different subsets of *AvrPm3* variants in wheat powdery mildew [49].

We also detected a common genomic region associated with virulence to another group of six *Pc* genes (Pc*38*/*39*/*55*/*63*/*70*/*71*). Leonard et al [50] previously detected a group including *Pc38*, *Pc39*, *Pc55*, *Pc63*, *Pc70* and *Pc71* for which the pathogen virulence phenotypes against a suite of North American and Israeli isolates of *Pca* were strongly associated. They suggested that the genes *Pc39*, *Pc55*, and *Pc71* may be independent identifications of the same resistance gene or alleles with identical race specificity. This is consistent with our findings that virulence to these genes maps to the same genomic location in *Pca*. They also detected another associated virulence group including *Pc45*, *Pc46*, *Pc48*, *Pc52*, *Pc54*, and *Pc57*, but not *Pc61*. This result partially overlaps with the *Pc48/52/57/61* group we identified here. The GWAS results may indicate that independent *R* genes have a redundant function by recognizing the same Avr effector, and/or that these are alleles of the same locus that have diversified to recognize variations of a single Avr effector. For the *Pc48* and *Pc38* groups, we detected two and three candidate effector sequences within the association interval, respectively. Thus, it is also possible that genes in these groups may recognize different but closely linked *Avr* genes. Notably, the *AvrSr35* and *AvrSr50* genes occur adjacent to each other separated by less than 15 kbp in the genome of *Pgt* [15]. While more work is needed to validate the function of candidate Avr effectors, these findings will assist future development of molecular markers for diagnosis of virulence.

Very few *Pc* genes have been mapped to the *A*. *sativa* consensus (Mrg) linkage groups and assigned to a chromosomal location [24,25,51,52]. For the group *Pc48*/*52*/*57*/*61* only *Pc48* has been mapped to *A*. *sativa* consensus linkage group Mgr20. For the group *Pc38*/*39*/*55*/*63*/*70*/*71*, previous data suggests that *Pc38* is allelic or linked to *Pc63* [53], while *Pc39* is linked or allelic to *Pc55* [54]. These results are consistent with our data where *Pc38* and *Pc63* show a very similar resistance profile, as do *Pc39* and *Pc55*. However, some of these genes have been assigned to different locations in the hexaploid oat consensus linkage map with *Pc38* mapped to the Mrg02 group [55], *Pc71* mapped to Mrg05 [26] and *Pc39* mapped to Mrg11 [56]. Thus, these distinct genes may show functional redundancy in recognizing effectors encoded at the same *Avr* locus. Given the extensive translocations in the oat genome [51,57] it is also possible that these sets of resistance genes represent an orthologous group that has been redistributed in the oat genome.

The temporal separation of our datasets allowed us to search for evidence of selective sweeps within the intervening interval [25,58]. Such signatures of positive selection can occur when an effector allele spreads in the population as it confers survival advantages to the pathogen by escaping host recognition. Our study detected several effector candidates that displayed such signatures shifting from high diversity in 1990 to reduced diversity in 2015. One of these candidate effectors showed an association with virulence on *Pc96* while others may correspond to *Pc* genes present in cultivated oat varieties that are not represented in the oat differential set. Most notably there was evidence for a selective sweep occurring at the *AvrPc38/39* locus associated with the evolutionary shift of the pathogen population to uniform virulence on these resistance genes by 2015.

This work demonstrates GWAS is a powerful tool to identify *Avr* gene candidates in sexual populations of rust fungi and also highlights the value of temporal comparisons to detect changes in virulence profiles and genetic diversity in pathogen populations over time. A population genomics approach allows inferences about population history and prediction of evolutionary potential. The persistent detection of recombination indicates a strong contribution of sexuality to the evolution of *Pca* in North America, consistent with the widespread distribution of the alternate host. This suggests that this pathogen can respond rapidly to changes in the environment such as the deployment of new resistance genes.

## Methods

### *Puccinia coronata* f. sp. *avenae* isolates, plant inoculations, and virulence phenotyping and analysis

*P*. *coronata* f. sp. *avenae* isolates were collected in 1990 and 2015 as part of the USDA-ARS Cereal Disease Laboratory annual rust surveys **(Table S1)** and are stored at -80°C as single-pustule cultures. Isolates were subjected to a second single-pustule purification and tested for purity as previously described [23,59]. For oat differential line [16,23] inoculations, urediniospores were heat shock activated (45°C, 10 minutes), placed in a rehydration chamber for at least 2.5 h, and then resuspended in Soltrol 170 oil (Chevron Phillips) at 2 mg spores/ml for spray-inoculation (750 μl per differential set). Inoculated plants were allowed to air dry and then placed in dew chambers for 16 hours under dark conditions with 10 minutes of continuous mist, followed by 40 seconds of misting every 2 minutes until the next day. Plants were removed from the growth chamber, allowed to air dry, and then placed in a greenhouse (22–25°C, 16 h photoperiod). Infection types were scored 10–12 days after inoculation, with a scale of “0”, “0;”, “;”, “;C”, “1;”, “1”, “2”, “3”,”3+”, and “4” [16]. At least two replicate inoculations were performed for all isolates. This scale was converted to a 0–9 numeric scale and mean values were obtained for heat map generation as previously described [16]. A Wilcoxon rank sum test was used to compare overall virulence of the 2019 and 2015 isolates across the entire oat differential set and that of the isolates per oat line in RStudio (1.3.959). Results were visualized using ggplot2 (v3.3.2) [60] Heatmaps were made from the numerical scoring data in R (3.4.0) [61] using ComplexHeatmap (v1.14.0) [62]. The R package pcaMethods (v1.68.0) [63] was used to perform principal component analysis of the numerical scoring data. Results were visualized with ggplot2 (v2.2.1) [60] and ggrepel (v0.8.0) [64].

### Buckthorn distribution analysis

Occurrence data by US county for *R*. *cathartica* (common buckthorn) was obtained from EDDMapS [40] on 05/18/2018 and plotted using ggplot2 (v2.2.1) in R (3.4.0) [60,61]. The locations of the 1990 and 2015 population collections were overlaid on the buckthorn distribution map. Geographic information for 1990 collection isolates was limited to the state level, except for those isolates derived from Minnesota Matt Moore buckthorn nursery (St. Paul, MN). Thus, the collection location was assigned to the state capital in order to generate the plot.

### DNA extraction from *P*. *coronata* f. sp. *avenae* urediniospores and Illumina sequencing

For DNA extraction, 5–10 mg of urediniospores were placed into a 2 ml screw-cap tube containing Lysing Matrix C (#116912100, MP Biomedicals) and 20–25 mg diatomaceous earth (#157607, MP Biomedicals). Samples were ground twice in a FastPrep-24™ 5G (#116005500, MP Biomedicals) for 20 seconds inverting tubes between grinds, at a speed setting of 4. Next, gDNA was extracted using the OmniPrep DNA Extraction Kit (#786–136, G-Biosciences). Briefly, 600 μl Genomic Lysis Buffer and 6.0 μl Proteinase K (both supplied with the kit) were added to the ground tissue, and samples were incubated at 60°C for 1 hour, mixing gently by inversion every 15–20 minutes. Subsequently, the manufacturer’s protocol for “Solid Tissue” was followed from step 5, and the optional 2 μl of Mussel Glycogen (supplied with the kit) was added as a DNA carrier in step 9. DNA was resuspended in Qiagen EB (#19086, Qiagen) instead of TE and the optional 1 ul of LongLife RNase (included with the kit) was added at step 13. TruSeq Nano Illumina DNA libraries with a 350-bp insert size were prepared from 100 ng of genomic DNA. Two batches of 30 libraries were multiplexed and sequenced in 3.5 lanes (HiSeq 2500, High Output Mode, 125 bp paired-end reads) at the University of Minnesota Genomics Center (UMGC) (MN, USA).

### Quality control of Illumina data, read mapping, and coverage assessment

Demultiplexed FASTQ files containing raw reads for each sample were subjected to Illumina adapter trimming using Trimmomatic (v0.33) [65] with the parameters ILLUMINACLIP 2:30:10. Adapter-trimmed FASTQ files were inspected with the quality control software FASTX-Toolkit (http://hannonlab.cshl.edu/fastx_toolkit/, 0.0.14), and quality and nucleotide distribution plots were generated and inspected for each library. Trimmed FASTQ files were aligned to the 12SD80 or 12NC29 primary contig reference genomes of *P*. *coronata* f. sp. *avenae* [22] with BWA-MEM (v0.7.15) [66] using default parameters. The SAM output from BWA-MEM was piped directly to SAMtools (v1.5) [28] for conversion to BAM format, sorting, PCR duplicate removal, and indexing. Summary statistics of read mapping were generated using SAMtools flagstat.

BEDtools (v2.25) [67] was used to generate mean mapping coverage for each isolate across each primary contig in the 12SD80 reference genome and distributions were visualized with ggplot2 (v2.2.1) [60] and ggridges (v0.5.0) [68] in R (3.4.0) [61]. To identify areas of missing coverage across the reference genome for each isolate, BEDtools makewindows was used to split the genome into 50 kbp-bins and then coverage across the bins was calculated using the *-hist* option. The resulting output was then analyzed in R to identify windows with a per-base depth of less than two for over 75% of the window and those were flagged as missing.

### Variant calling and annotation

Three different software packages were used to identify variants and determine which caller was most robust. First, variants were called jointly for all isolates in a given year with FreeBayes (v1.1.0) [27] with default parameters. Secondly, the Genome Analysis Toolkit (GATK v3.7.0) HaplotypeCaller [69] was used to call variants for all 60 isolates independently with the parameters—*genotyping_mode DISCOVERY—emitRefConfidence GVCF*, and then GenotypeGVCFs was run to perform joint variant calls for 1990 and 2015 separately with default parameters. Thirdly, SAMtools (v1.5) mpileup and BCFtools (v1.6) [28] in multiallelic calling mode were used to call variants. FreeBayes produced the most robust variant calls, since it gave the highest fraction of calls supported by all three algorithms (**S16 Fig**) and SNP data from this algorithm was used in subsequent analyses. To accurately compare variants from the three callers, all resulting vcf files were quality filtered with vcffilter in vcflib (v1.0.0-rc1, commit 717cfbf) with the parameters *QUAL > 20* & *AC > 0*, and then the representations of the variants were regularized by using *vcfallelicprimitives* in vcflib as representations of variants that differ between methods (68). Finally, *vcfintersect* in vcflib was used to obtain variants in common and unique to each caller. UpSet diagrams to visualize comparisons were made with the R package UpSetR (v1.3.3) [70].

To further characterize shared and unique variants to each caller, BEDtools (v2.25) [67] annotate was used to count variants overlapping genes and repeats (**S17 Fig**). Prior to annotation, start and stop coordinates for genes and repeats were merged with BEDtools merge if they overlapped. To analyze GC content surrounding variants, BEDtools slop was used to make 1 kbp-windows downstream and upstream of each variant, and then BEDtools nuc was used to extract GC content of these windows. Percentages of shared and unique variants overlapping gene and repeat coordinates, as well as percent GC in windows surrounding variants, were then plotted with ggplot2 (v2.2.1) in R (3.4.0) (**S17 Fig**) [60,61].

To generate the final FreeBayes vcf files for all subsequent downstream analysis, SNPs were filtered with vcffilter in vcflib (v1.0.0-rc1, commit 717cfbf) with the parameters *QUAL > 20* & *QUAL / AO > 10* & *SAF > 0* & *SAR > 0* & *RPR > 1* & *RPL > 1* & *AC > 0*. To compare variants between 1990 and 2015 isolates, *vcfintersect* in vcflib was used as described above. Statistics were generated with vcfstats in vcflib, and UpSetR (v1.3.3) [70] was used to visualize shared and unique variants between years. The functional impact of variants, e.g. nonsynonymous or synonymous, was annotated using ANNOVAR (version 2017Jul16) [71]. The R package vcfR (v1.8.0) [72] was used to manipulate vcf files for calculations of allele balance to confirm single fungal genotypes free of contamination. The vcfR functions *extract*.*gt* and *is_het* were used to extract heterozygous positions. Those with allele depths that were outside of the 0.15 and 0.95 quantiles were removed as these are likely lower confidence variants due to insufficient sequence coverage. Distributions of allele balances were plotted using ggplot2 (v2.2.1) [60].

### Population structure, phylogenies, and genetic differentiation

The *k*-mer Weighted Inner Product (kWIP) [30] was used to measure genetic relatedness between isolates. A hash table of mapped reads was generated with khmer (v2.1.1) [73,74] using the parameters *-N 1 -k 20 -b -T 24 -f -s tsv -M 100e9*, and then kWIP was used to calculate the weighted distance matrix between all isolates for each year and combined. The R script provided with kWIP was slightly modified to perform classical multidimensional scaling (MDS) with the cmdscale function of the resulting distance matrix. Ggplot2 (v2.2.1) [60] was used for visualization.

The R package adegenet (v2.1.1) [31] was used to perform a Discriminant Analysis of Principal Components (DAPC). vcfR (v1.8.0) [72] was used to import and convert vcf files to genlight objects. After conversion there were 1,379,292, 1,186,114, and 2,007,463 biallelic SNPs retained for the 1990, 2015, and combined year datasets, respectively. Prior to running DAPC, the optim.a.score function was used to identify the optimal number of principal components to retain for each dataset (1990, 10; 2015, 9; combined years, 20). Three discriminant components were retained for all datasets. Results were visualized using the scatter and compoplot functions in adegenet.

Phylogenetic analysis of isolates was performed using the maximum likelihood criterion (ML) in RAxML (v8.2.11) (74). vcfR (v1.8.0) (71) [72] was used to convert vcf files to PHYLIP and NEXUS formats. Support for groups was assessed using 500 bootstrap replicates and a general time reversible (GTR) model. Results were visualized using the ggtree R package (v1.8.2) [75]. Pairwise *F*_ST_ values were estimated between years and subgroups within each year using the PopGenome R package (v2.6.1) [34,76].

To infer on the occurrence of reticulation in *Pca*, we reconstructed an unrooted phylogenetic network in SplitsTree (v4.15.1) [77]. We used the dataset of our collection of 60 isolates mapped against 12SD80 reference strain and visualized the final network in iTOL (v5.6.3) [78]. Inferences were also performed individually within each sampling year. The necessary steps for file conversion were fulfilled with a custom python script [79]. We next tested for the signature of past recombination events by performing a PHI-test in SplitsTree [33,77].

### Linkage disequilibrium

We used the standardized index of association (*r_d_*) (78) as implemented in the R package poppr (v2.8.0) [80,81] to measure multilocus LD in 1990 and 2015 isolates. We used the sample.ia function from poppr to calculate *r_d_* for 100 sets of 10,000 random SNPs to generate a distribution of values. The distributions for each year were compared to the distributions of 10,000 *r_d_* values constructed using simulated datasets with 0, 50, 75, and 100% linkage as done previously [36]. The adegenet package in R was used to conduct the simulations of a diploid dataset containing 1,282,703 loci (average between number of loci in 1990 and 2015 datasets) and 30 samples. A Kruskal-Wallis rank sum test was used to compare across the observed and simulated *r_d_* distributions using the kruskal.test R function, and then the kruskal function in the R package agricolae (v1.2.8) [82] was used to compare means of the ranks using a Bonferroni correction for *p*-adjustment for multiple comparisons.

### Selection and nucleotide diversity analyses

The PopGenome R package (v2.6.1) [76] was used to calculate population genomic statistics including nucleotide diversity per site (*π*) [34,83], Watterson’s *θ* [84], and Tajima’s *D* [85] for each year and subpopulations within years. All calculations were performed on a per-contig genome-wide level and for all genes and effectors [22]. Statistical comparisons between years, subpopulations, or gene sets were made using Wilcoxon rank sum tests with the wilcox.test function in R. The composite likelihood ratio (CLR) [37] selective sweep statistic was calculated using PopGenome to test for selective sweeps in effectors. Summary plots for all population genomic statistics were generated using ggplot2 (v2.2.1) [60].

### Presence/Absence variation analysis of effector loci

Summary statistics computed with PopGenome were compiled for all effector genes and plotted with ggplot2 (v2.2.1) [60]. Presence/absence variation (PAV) was measured in effectors using BEDtools (v2.25) [67] coverage with the -hist option to get per-base coverage for each gene. Using the per-base coverage output from BEDtools, the percentage of base-pairs for which the coverage was less than five reads was then calculated for each gene. ComplexHeatmap (v1.14.0) [62] was used to plot summary heatmaps of the percent missing for each effector. The R package ggbio (v1.24.1) [86] was used to visualize mapping coverage across genomic regions. To search for homology to candidate effectors showing PAV and evidence of selective sweeps, BLASTp was used against a custom database of effectors from *Puccinia striiformis* f. sp. *tritici* [87] and *Puccinia graminis* f. sp. *tritici* [88]. Canonical Correspondence Analysis (CCA) [89] was conducted using the R package vegan (v2.5–3) and results were visualized with ggvegan (v0.0–9) [90].

### Genome-Wide Association Study (GWAS)

SNPs were called separately against 12SD80 and 12NC29 primary contig reference genomes [22] using reads from 62 isolates (60 isolates plus 12SD80 and 12NC29). The biallelic SNP datasets generated by FreeBayes were filtered for SNPs scored on at least 90% of the isolates and with >5% minor allele frequency (MAF). From 1,595,920 biallelic SNPs for 12SD80 and 1,604,163 for 12NC29, 860,621 and 833,042 SNPs passed the filtering for 12SD80 and 12NC29, respectively. The Trait Analysis by aSSociation, Evolution and Linkage (TASSEL) software (v5.0) [91] was used for the GWAS, and an additional default filtering at >5% MAF was imposed upon importing the genotype data (vcf format), resulting in a final SNP count of 848,886 for 12SD80 and 821,193 for 12NC29. In TASSEL, the population structure and kinship were calculated from the genotype data using four principal components and centered IBS, respectively, as inputs for a Mixed Linear Model (MLM) workflow, together with the genotype and the phenotype (0–9 scale) files. For the marker-trait associations, no compression option was selected and variance components were re-estimated after each marker. Marker-trait associations were conducted for all oat differential lines. To validate, a subset of the data was run in MLM in R using the package GAPIT (Genome Associated Prediction Integrated Tool) (v3) [91–93]. The MLM statistics from TASSEL were exported to create customizable Manhattan plots in R using the qqman package (v0.1.4) [93]. To declare significant SNPs, the qvalue package (v2.15.0) in R [94] was used to compute the false discovery rate (FDR) at 5%. The Bonferroni correction threshold was calculated by dividing the p value (α = 0.05) with the number of markers used in GWAS, resulting in a threshold of 5.89 x 10^−8^ for 12SD80 and 6.09 x 10^−8^ for 12NC29. Synteny between contigs was determined by D-Genies alignment [95] and homology between candidate effectors was deduced by aligning two or more sequences in BlastN using default parameters.

## Supporting information

S1 Fig Principal component analysis of *Puccinia coronata* f. sp. *avenae* isolate virulence phenotypes. **(A), (B)** and **(C)** Plots show PC1 (x-axis) and PC2 (y-axis) scores and loadings for isolates from both years. **(D)** and **(E)** Plots show PC1 (x-axis) and PC2 (y-axis) scores and loadings for isolates from 1990. **(F)** and **(G)** Plots show PC1 (x-axis) and PC2 (y-axis) scores and loadings for isolates from 2015.
(TIF)

S2 Fig Read mapping coverage of *Puccinia coronata* f. sp. *avenae* isolates. Plot shows the distribution of average read mapping coverage for all primary contigs of the 12SD80 isolate reference genome for each sequenced isolate.
(TIF)

S3 Fig Allele balance at heterozygous positions to assess genotype contamination. **(A)** Distributions of the frequencies of the most abundant allele for heterozygous positions (dark blue) and the frequencies of the second most abundant allele (light blue) for 1990 isolates. **(B)** Distributions of the frequencies of the most abundant allele for heterozygous positions (dark blue) and the frequencies of the second most abundant allele (light blue) for 2015 isolates. Isolate name is provided above each distribution.
(TIF)

S4 Fig Genetic relatedness of *Puccinia coronata* f. sp. *avenae* isolates. **(A)** kWIP Genetic distance matrix of combined isolates from 1990 and 2015 collections. **(B)** Genetic distance matrix of isolates sampled in 1990. **(C)** Genetic distance matrix of isolates sampled in 2015. Geographic origin of isolates is indicated by color key.
(TIF)

S5 Fig Population differentiation of *Puccinia coronata* f. sp. *avenae* isolates. **(A)** DAPC results using two populations defined by each year (1990 in black and 2015 in red). The left panel shows the densities of individuals on the single discriminant function and the right panel shows the membership probabilities. **(B)** Scatterplot (upper panel) and membership probabilities (lower panel) from discriminant analysis of principal components between the subpopulations (see colored key). The ellipses represent a summary of the isolates in each population with the X marking the center of the data cloud. Geographic origin of isolates is indicated by color key. The thick horizontal colored bars in lower part the membership plots represent the geographic origin of the isolates.
(TIF)

S6 Fig Genetic differentiation and linkage disequilibrium in *P*. *coronata* f. sp. *avenae* subpopulations. **(A)** Distribution of *F*_ST_ values between 1990 and 2015 for all contigs in the reference genome. Lines in the violin plot represent quartiles. **(B)** Pairwise *F*_ST_ values between subpopulations in each year. **(C)** Boxplot shows the observed *r_d_* distributions for 1990 (black) and 2015 (red) populations compared to the distributions of *r_d_* values for simulated datasets with 0, 50, 75, and 100% linkage (grey).
(TIF)

S7 Fig GWAS for virulence of *Pca* towards oat differential lines *Pc38*, *Pc39*, *Pc55*, *Pc63*, *Pc70* and *Pc71* (contig 134F and 250F). Red and blue horizontal lines denote Bonferroni significance threshold (α = 0.05/total number of markers) and 5% false discovery rate threshold, respectively. Highlighted SNPs in orange are derived from contigs with significant association peaks and containing predicted effector genes, whereas SNPs in purple are derived from significant contigs without any predicted effector genes. Note that contig 174F in 12SD80 was colored in orange as it contains a gene sequence with orthology to one in 12NC29 contig 268F encoding a predicted secreted protein.
(TIF)

S8 Fig Quantile-quantile (Q-Q) plots of GWAS in Fig 3. (TIF)

S9 Fig Quantile-quantile (Q-Q) plots of GWAS in Fig 4. (TIF)

S10 Fig Quantile-quantile (Q-Q) plots of GWAS in S7 Fig. (TIF)

S11 Fig Dot plots to illustrate alignment of synthenic contigs from 12NC29 and 12SD80. Color key indicates sequence identity ratios for all dot plots.
(TIF)

S12 Fig Single-contig Manhattan plots for contig 134F in 12SD80 and contig 310F in 12NC29. Significant SNPs above the FDR (0.05) are highlighted in orange. The positions of predicted effectors are shown in the gray bar, which represents the full-length contig. There are no predicted effectors in the orthologous contigs 566F and 250F.
(TIF)

S13 Fig Virulence phenotype heatmap of the 62 isolates on *Pc38*, *Pc39*, *Pc55*, *Pc63*, *Pc70* and *Pc71*. Infection scores were converted to a numeric scale (0 = resistance to 9 = susceptibility) for heatmap generation.
(TIF)

S14 Fig Genetic diversity and selection in *P*. *coronata* f. sp. *avenae*. **(A)** Distribution of nucleotide diversity (*π*) values for all genes and predicted effectors of 12SD80 using variants from 1990 and 2015 populations. **(B)** Distribution of Watterson’s *θ* values for all genes and predicted effectors of 12SD80 using variants from 1990 and 2015 populations. **(C)** Distribution of Tajima’s *D* values for all genes and predicted effectors of 12SD80 using variants from 1990 and 2015 populations. **(D)** Distribution of Fst values for all genes and predicted effectors of 12SD80 using variants from 1990 and 2015 populations. **(E)** Distribution of nucleotide diversity (*π*) values for contigs in the reference genome 12SD80 using variants from 1990 and 2015 collections. **(F)** Distribution of Watterson’s *θ* values for contigs in the reference genome 12SD80 using variants from 1990 and 2015 collections. **(G)** Distribution of Tajima’s *D* values for contigs in the reference genome 12SD80 using variants from 1990 and 2015 collections. Values in **A** and **B** were transformed as indicated on the y-axes to aid in visualization. Lines in the violin plots represent quartiles. *p*-values were calculated using the Wilcoxon rank-sum test.
(TIF)

S15 Fig Canonical correspondence analysis of the influence of presence/absence variation on phenotypic variation of *Puccinia coronata* f. sp. *avenae* isolates on the oat differential set. In the ordination plot, the black arrows show the contribution of the presence/absence variation of particular genes to the CCA axes. The PCASD_ prefixes were left off for clarity. The names in red are the oat lines in the differential set. CCA1 explained 44.9% of the total inertia and CCA2 explained another 15.6% of the total inertia.
(TIF)

S16 Fig Comparison of the performance of three variant callers. **(A)** SNPs unique to and shared between the three callers in the 1990 isolates. **(B)** SNPs unique to and shared between the three callers in the 2015 isolates. **(C)** INDELs unique to and shared between the three callers in 1990. **(D)** INDELs unique to and shared between the three callers in 2015.
(TIF)

S17 Fig Association of variants with genes and repeats as well as high GC content genomic regions. **(A)** Percent overlap of shared and unique variants with genes and repeats for 1990 and 2015 isolate datasets. **(B)** GC percent in 1 kbp windows surrounding shared and unique variants for 1990 and 2015 isolate datasets according to variant caller.
(TIF)

S1 Table Isolate locations, sequencing statistics, and FreeBayes variant calls in each isolate (excel file). (XLSX)

S2 Table Change in virulence in the oat differential set from 1990 to 2015 (excel file). (XLSX)

S3 Table Mean values of nucleotide diversity and neutrality in all genes and effectors. (DOCX)

S4 Table Population genetic calculations for all predicted effectors in the 12SD80 genome reference (excel file). (XLSX)

S5 Table Identified presence/absence variation and selective sweep effector candidates (excel file). (XLSX)

S6 Table Primary contigs in the 12SD80 genome reference with negative Tajima’s *D* values (excel file). (XLSX)
