# Supplementary figures and images for: Increased virulence of Puccinia coronata f. sp.avenae populations through allele frequency changes at multiple putative Avr loci
Source: PLoS Genet. 2020 Dec 28;16(12):e1009291. doi: 10.1371/journal.pgen.1009291 (PMC7793281; doi:10.1371/journal.pgen.1009291)

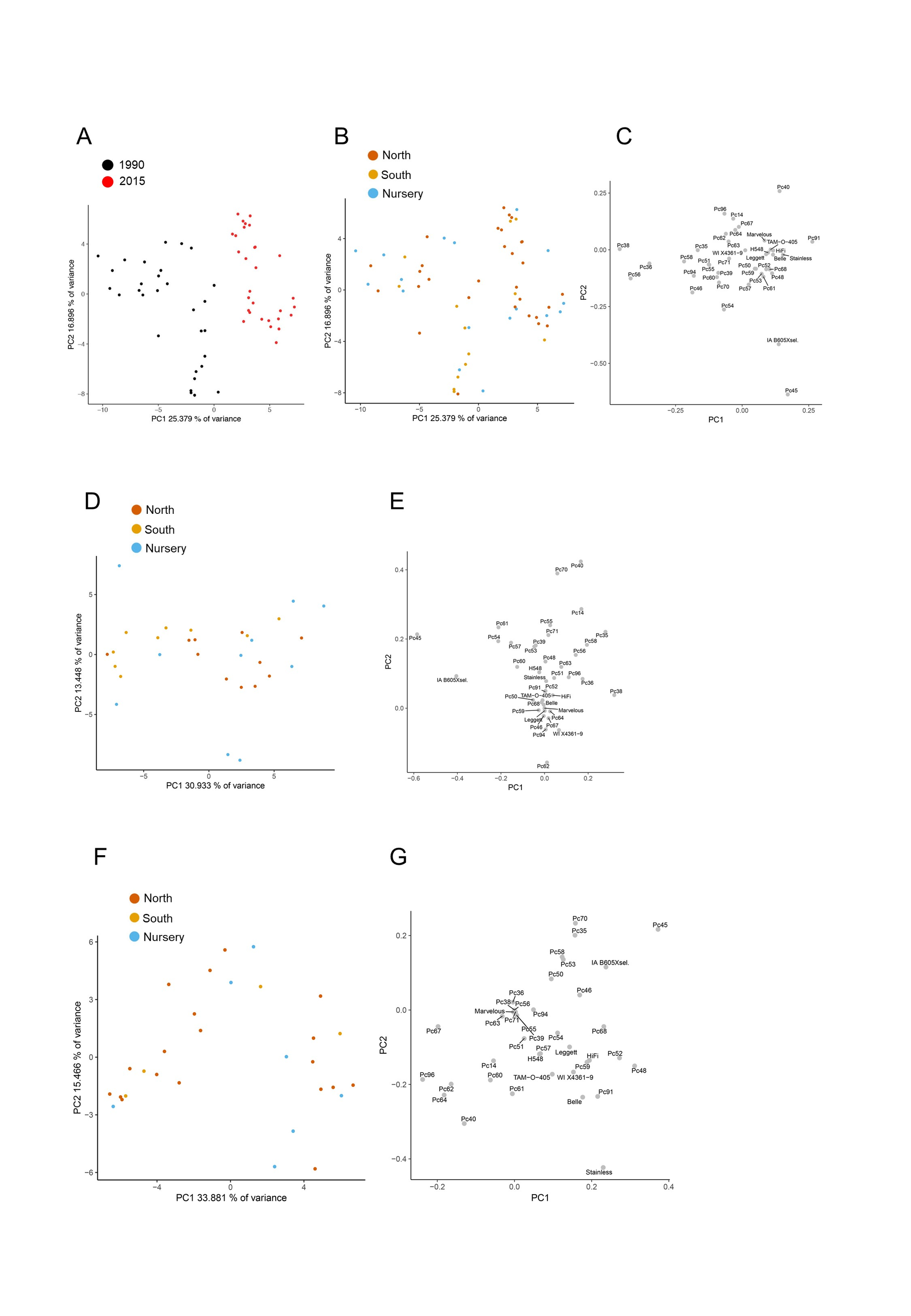

Supplement: S1 Fig — (A), (B) and (C) Plots show PC1 (x-axis) and PC2 (y-axis) scores and loadings for isolates from both years. (D) and (E) Plots show PC1 (x-axis) and PC2 (y-axis) scores and loadings for isolates from 1990. (F) and (G) Plots show PC1 (x-axis) and PC2 (y-axis) scores and loadings for isolates from 2015. (TIF) [file pgen.1009291.s001.tif]

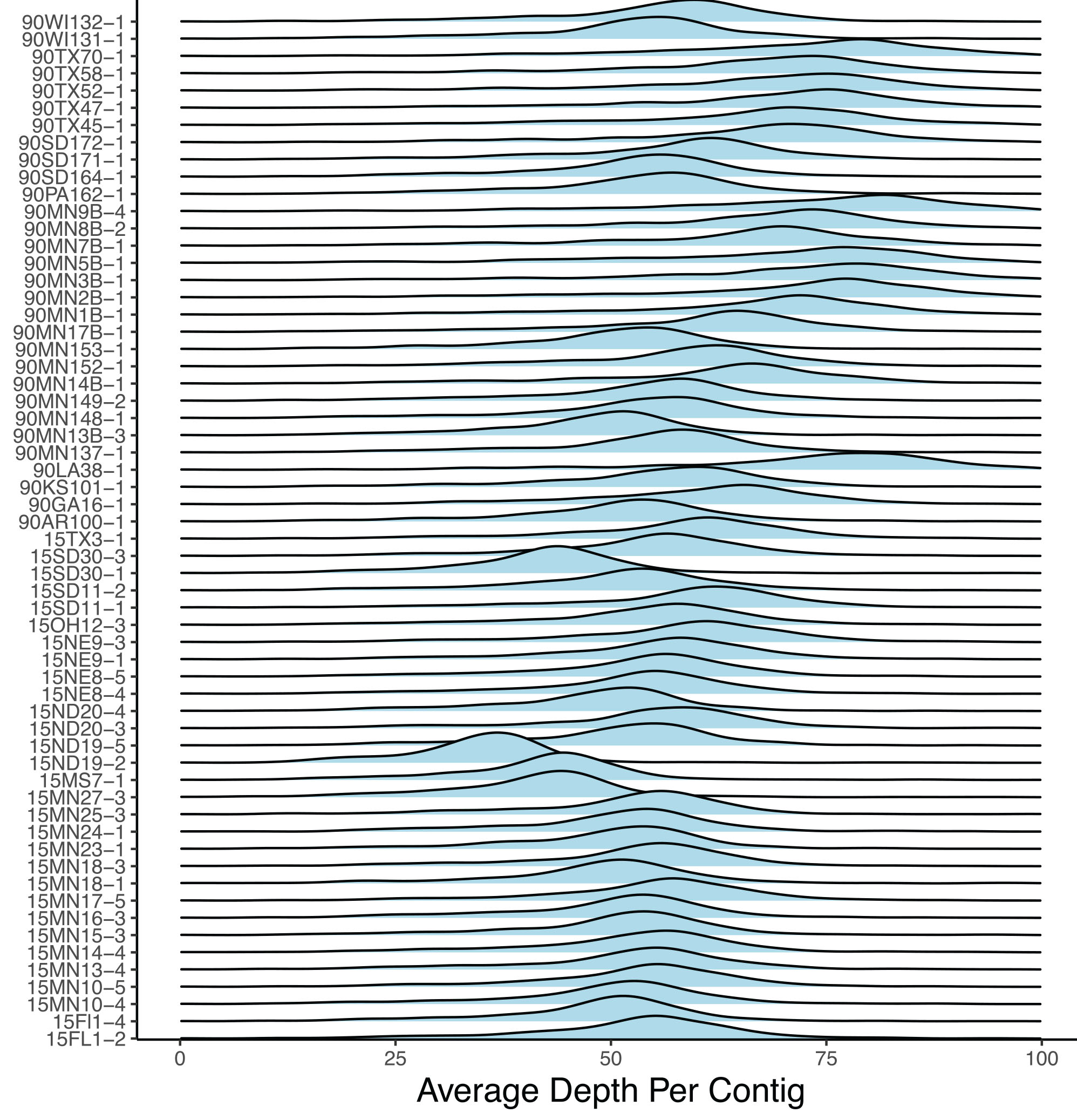

Supplement: S2 Fig — Plot shows the distribution of average read mapping coverage for all primary contigs of the 12SD80 isolate reference genome for each sequenced isolate. (TIF) [file pgen.1009291.s002.tif]

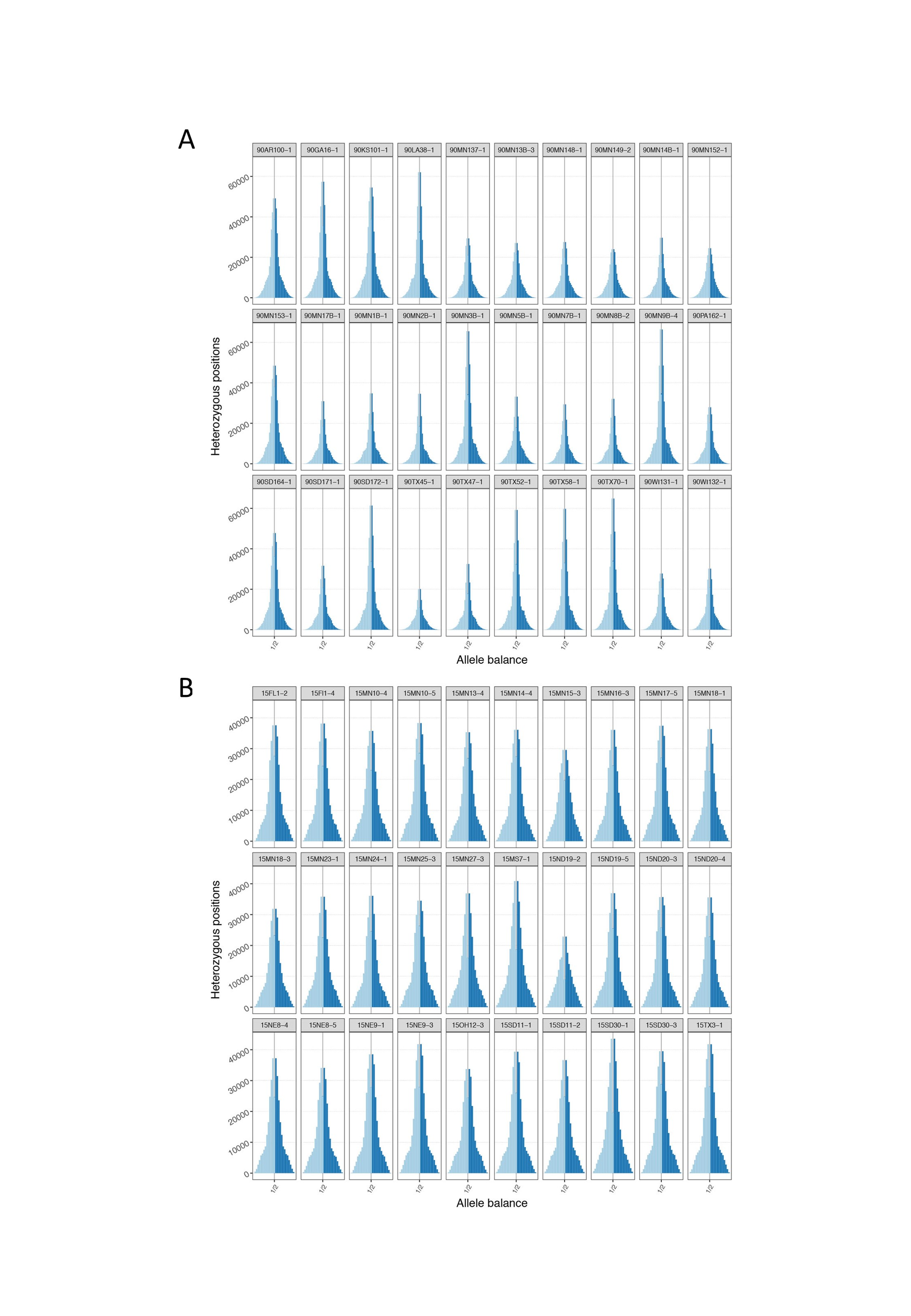

Supplement: S3 Fig — (A) Distributions of the frequencies of the most abundant allele for heterozygous positions (dark blue) and the frequencies of the second most abundant allele (light blue) for 1990 isolates. (B) Distributions of the frequencies of the most abundant allele for heterozygous positions (dark blue) and the frequencies of the second most abundant allele (light blue) for 2015 isolates. Isolate name is provided above each distribution. (TIF) [file pgen.1009291.s003.tif]

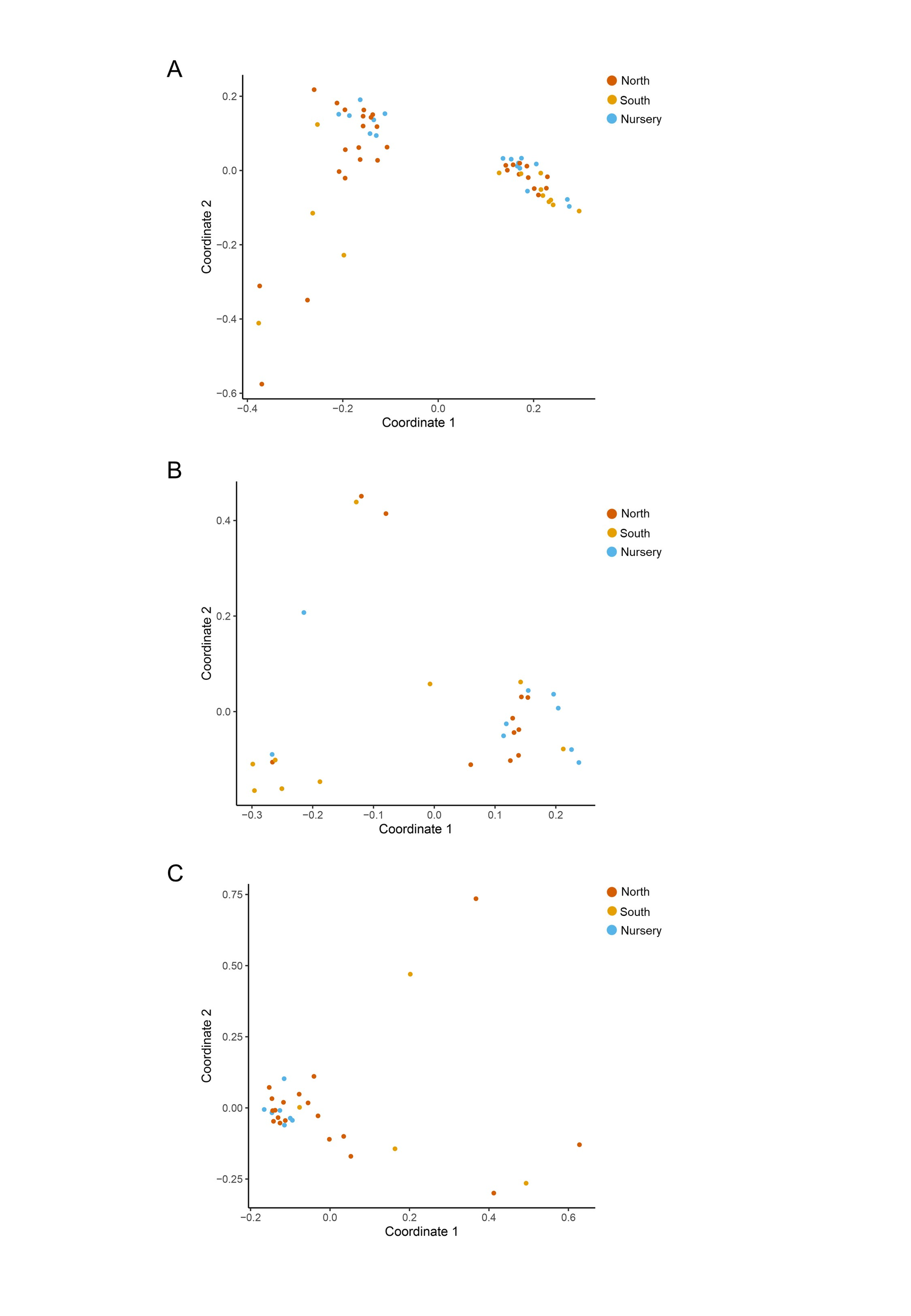

Supplement: S4 Fig — (A) kWIP Genetic distance matrix of combined isolates from 1990 and 2015 collections. (B) Genetic distance matrix of isolates sampled in 1990. (C) Genetic distance matrix of isolates sampled in 2015. Geographic origin of isolates is indicated by color key. (TIF) [file pgen.1009291.s004.tif]

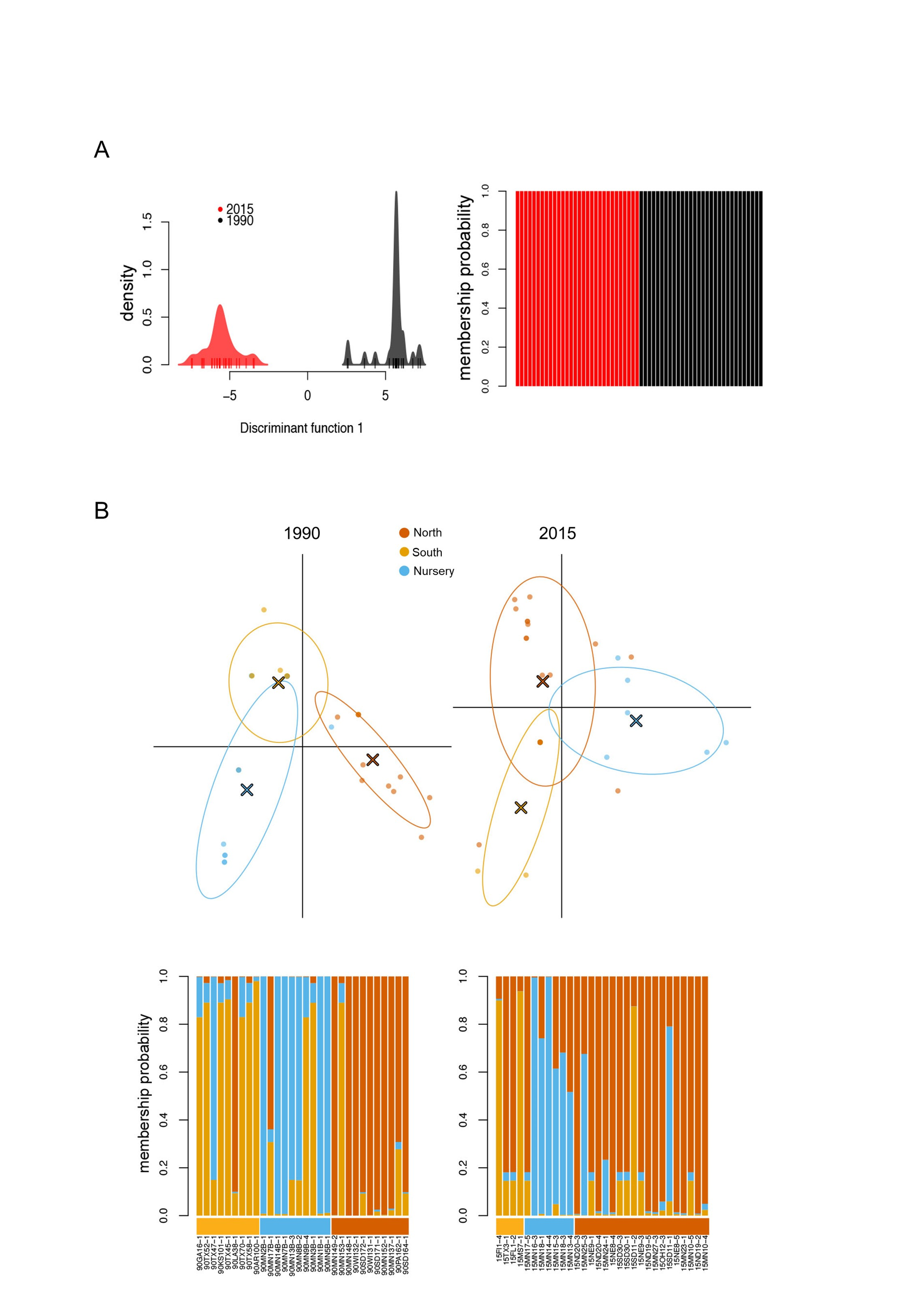

Supplement: S5 Fig — (A) DAPC results using two populations defined by each year (1990 in black and 2015 in red). The left panel shows the densities of individuals on the single discriminant function and the right panel shows the membership probabilities. (B) Scatterplot (upper panel) and membership probabilities (lower panel) from discriminant analysis of principal components between the subpopulations (see colored key). The ellipses represent a summary of the isolates in each population with the X marking the center of the data cloud. Geographic origin of isolates is indicated by color key. The thick horizontal colored bars in lower part the membership plots represent the geographic origin of the isolates. (TIF) [file pgen.1009291.s005.tif]

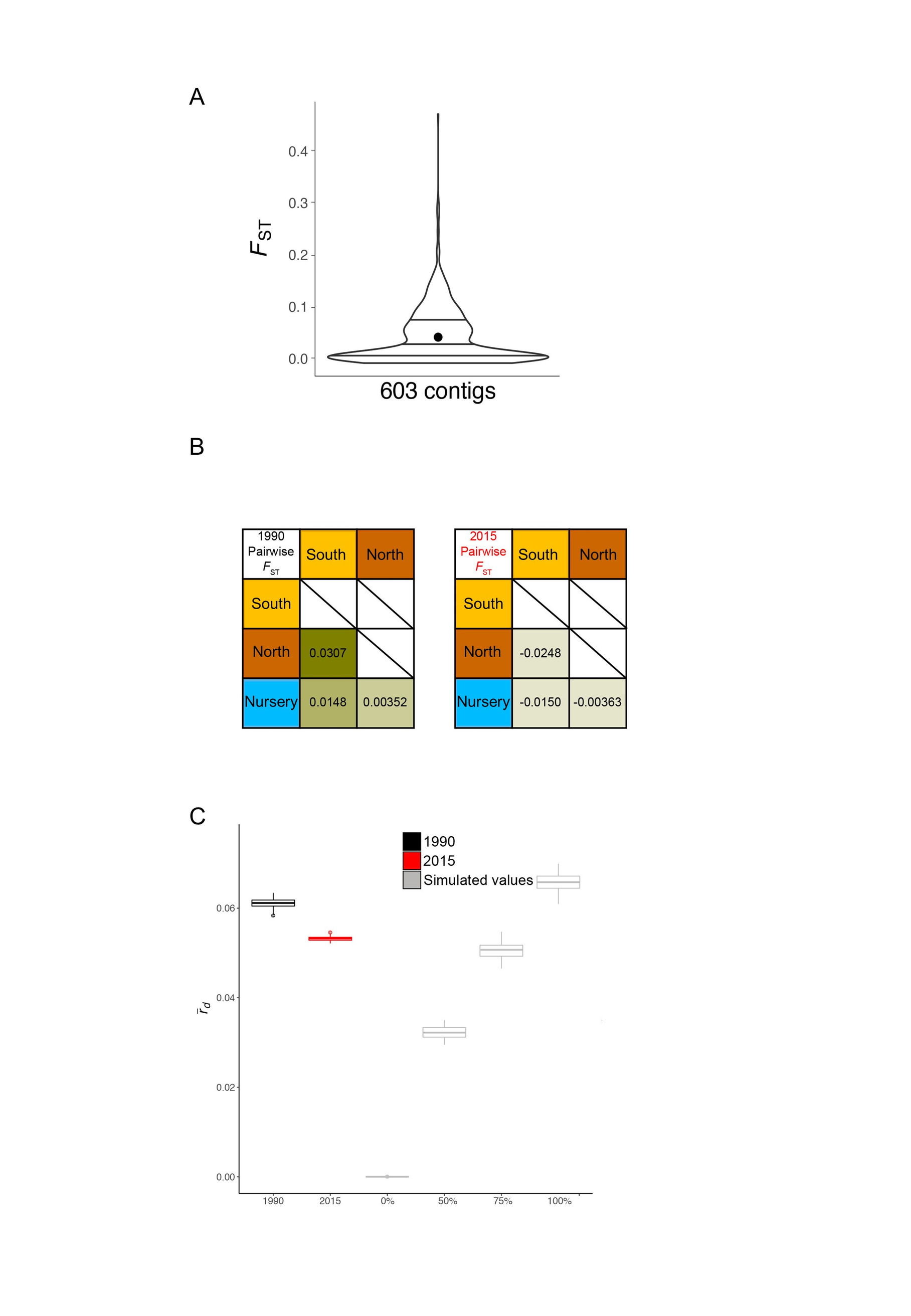

Supplement: S6 Fig — (A) Distribution of FST values between 1990 and 2015 for all contigs in the reference genome. Lines in the violin plot represent quartiles. (B) Pairwise FST values between subpopulations in each year. (C) Boxplot shows the observed rd distributions for 1990 (black) and 2015 (red) populations compared to the distributions of rd values for simulated datasets with 0, 50, 75, and 100% linkage (grey). (TIF) [file pgen.1009291.s006.tif]

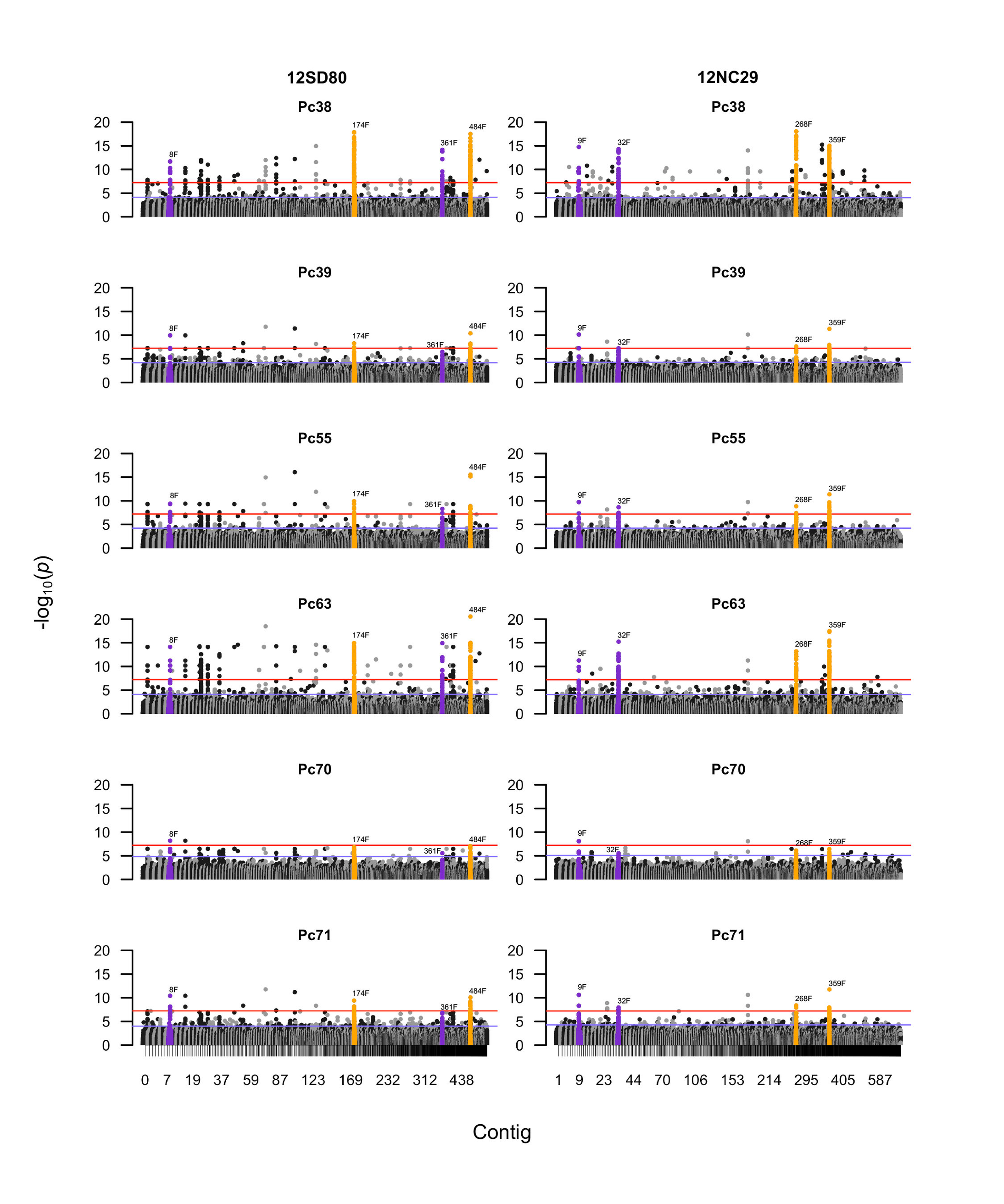

Supplement: S7 Fig — Red and blue horizontal lines denote Bonferroni significance threshold (α = 0.05/total number of markers) and 5% false discovery rate threshold, respectively. Highlighted SNPs in orange are derived from contigs with significant association peaks and containing predicted effector genes, whereas SNPs in purple are derived from significant contigs without any predicted effector genes. Note that contig 174F in 12SD80 was colored in orange as it contains a gene sequence with orthology to one in 12NC29 contig 268F encoding a predicted secreted protein. (TIF) [file pgen.1009291.s007.tif]

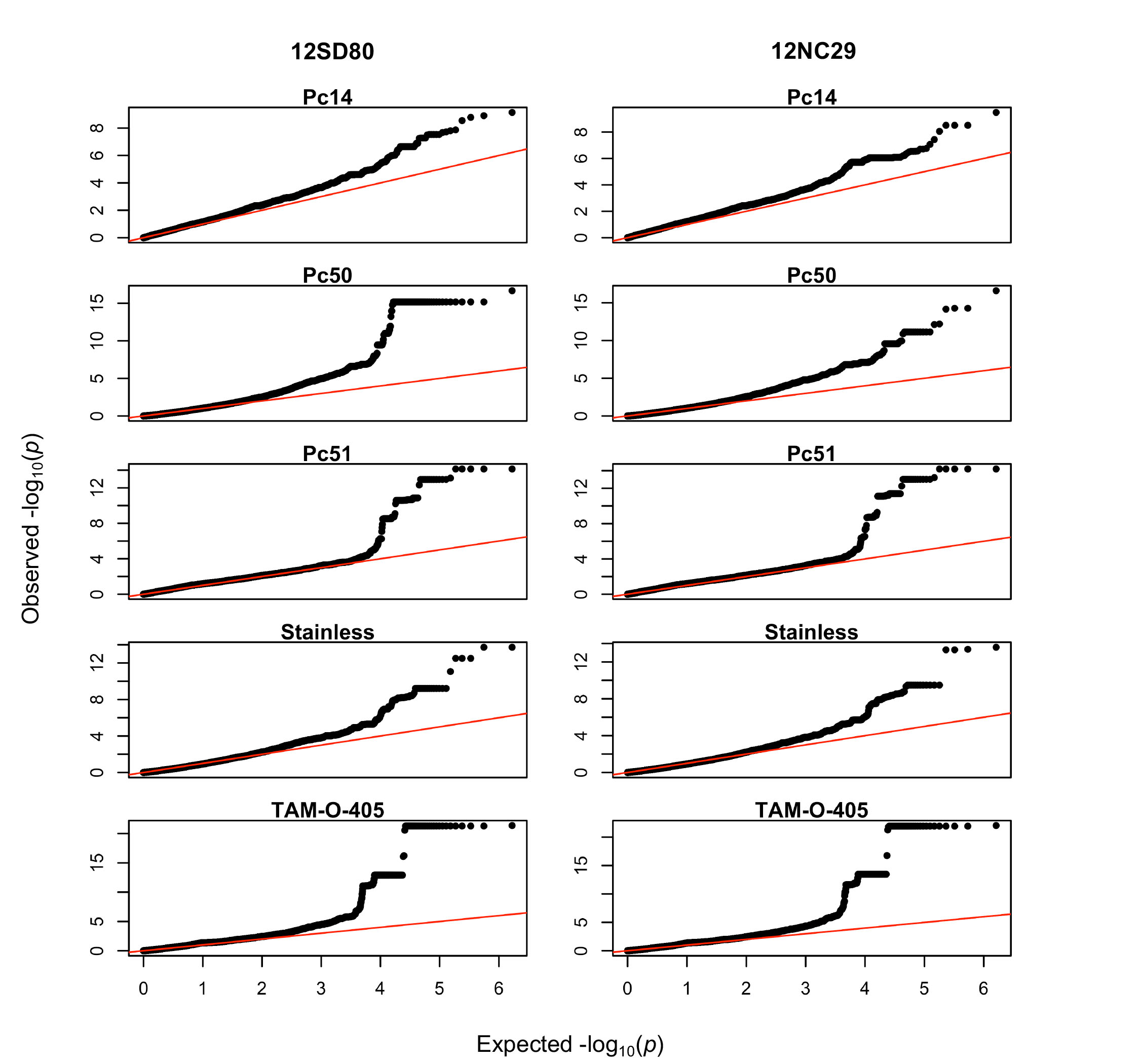

Supplement: S8 Fig — (TIF) [file pgen.1009291.s008.tif]

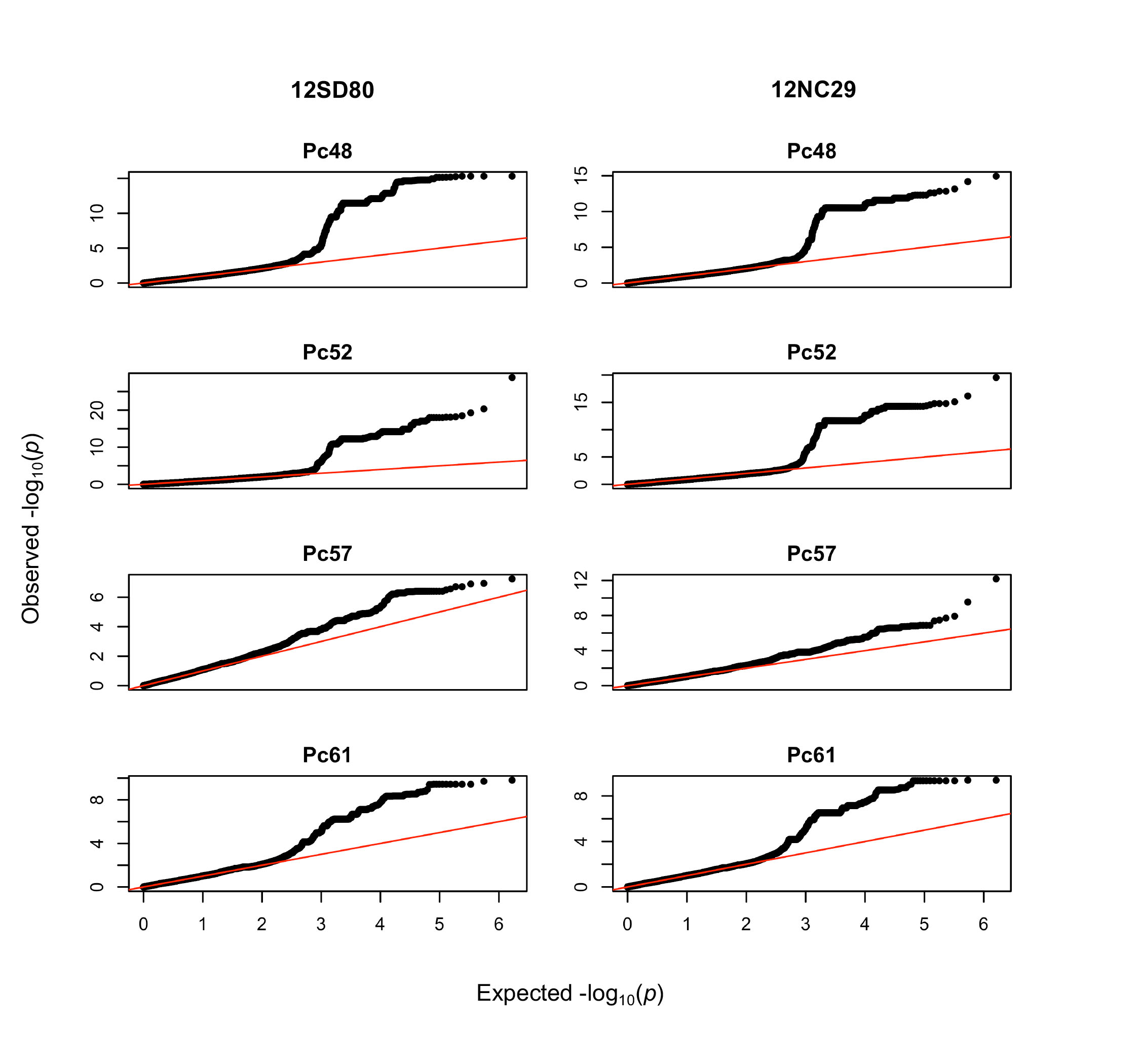

Supplement: S9 Fig — (TIF) [file pgen.1009291.s009.tif]

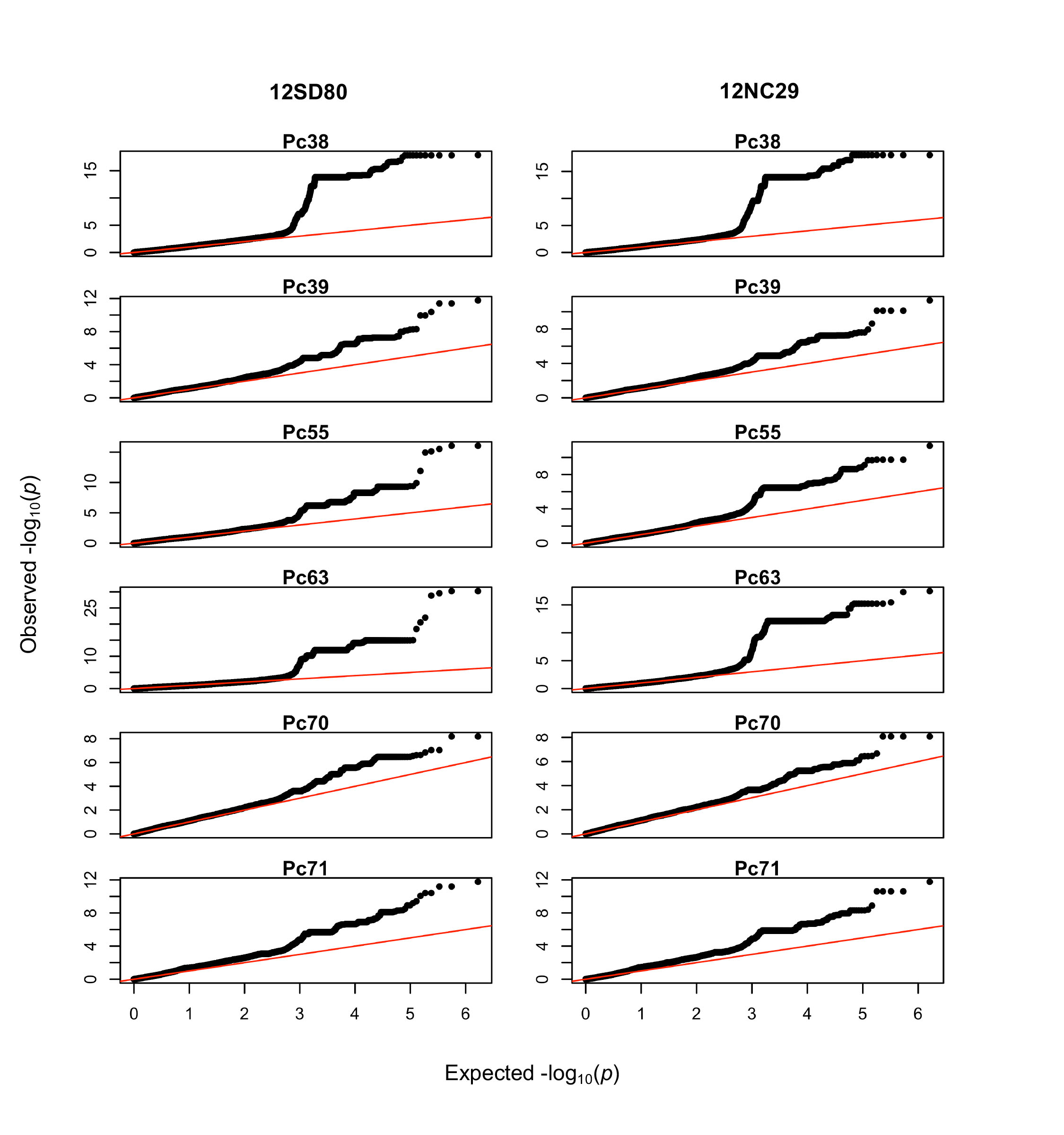

Supplement: S10 Fig — (TIF) [file pgen.1009291.s010.tif]

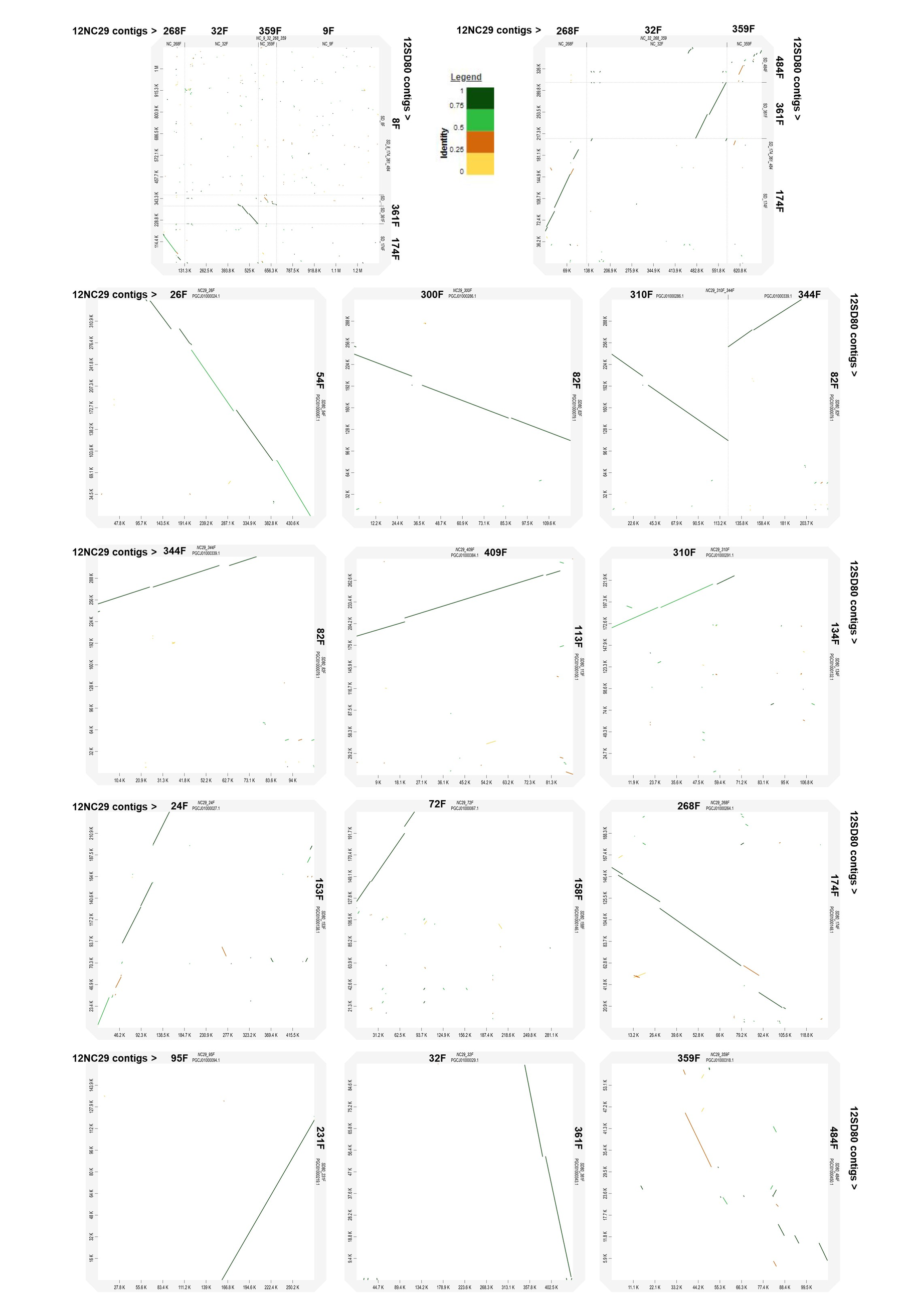

Supplement: S11 Fig — Color key indicates sequence identity ratios for all dot plots. (TIF) [file pgen.1009291.s011.tif]

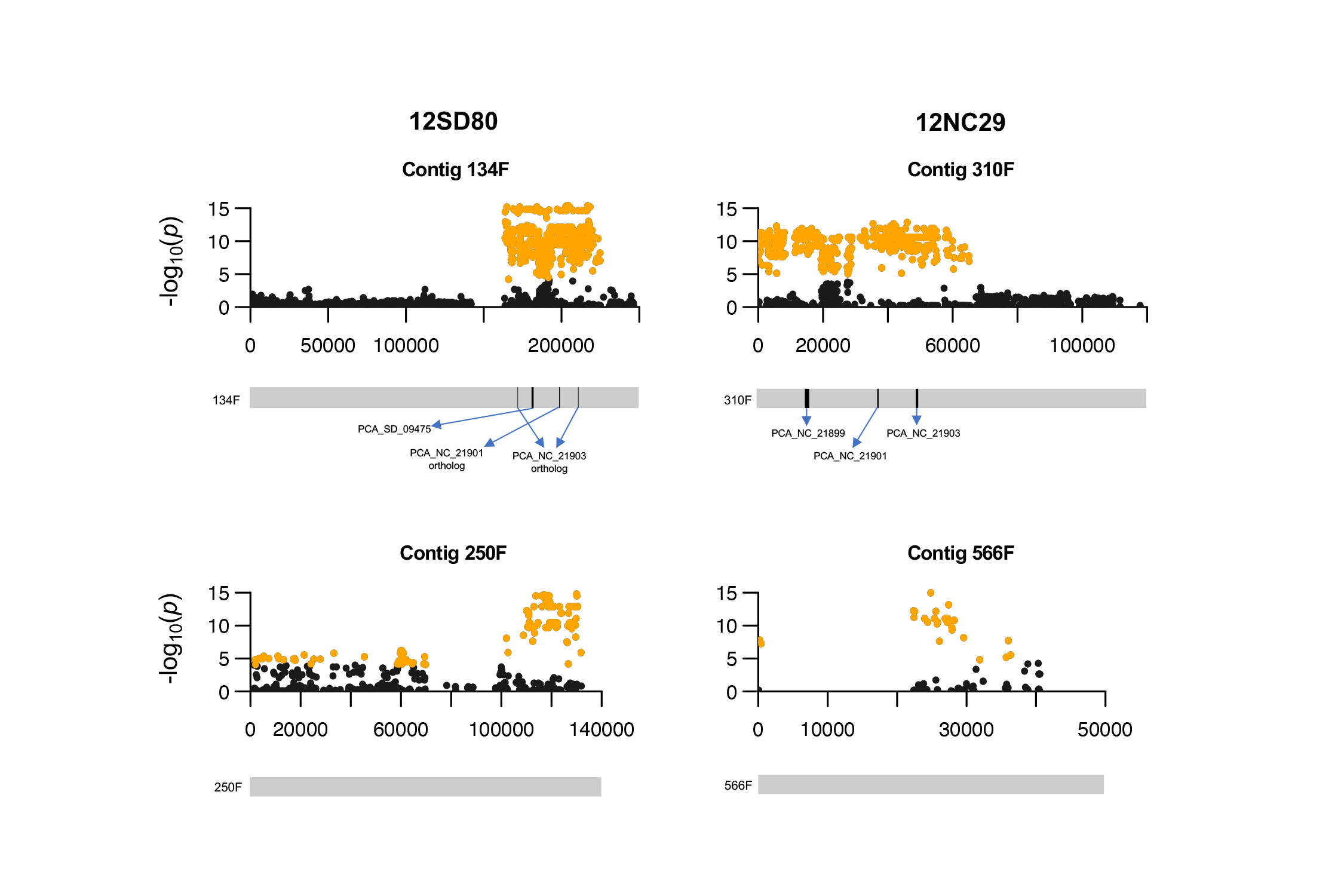

Supplement: S12 Fig — Significant SNPs above the FDR (0.05) are highlighted in orange. The positions of predicted effectors are shown in the gray bar, which represents the full-length contig. There are no predicted effectors in the orthologous contigs 566F and 250F. (TIF) [file pgen.1009291.s012.tif]

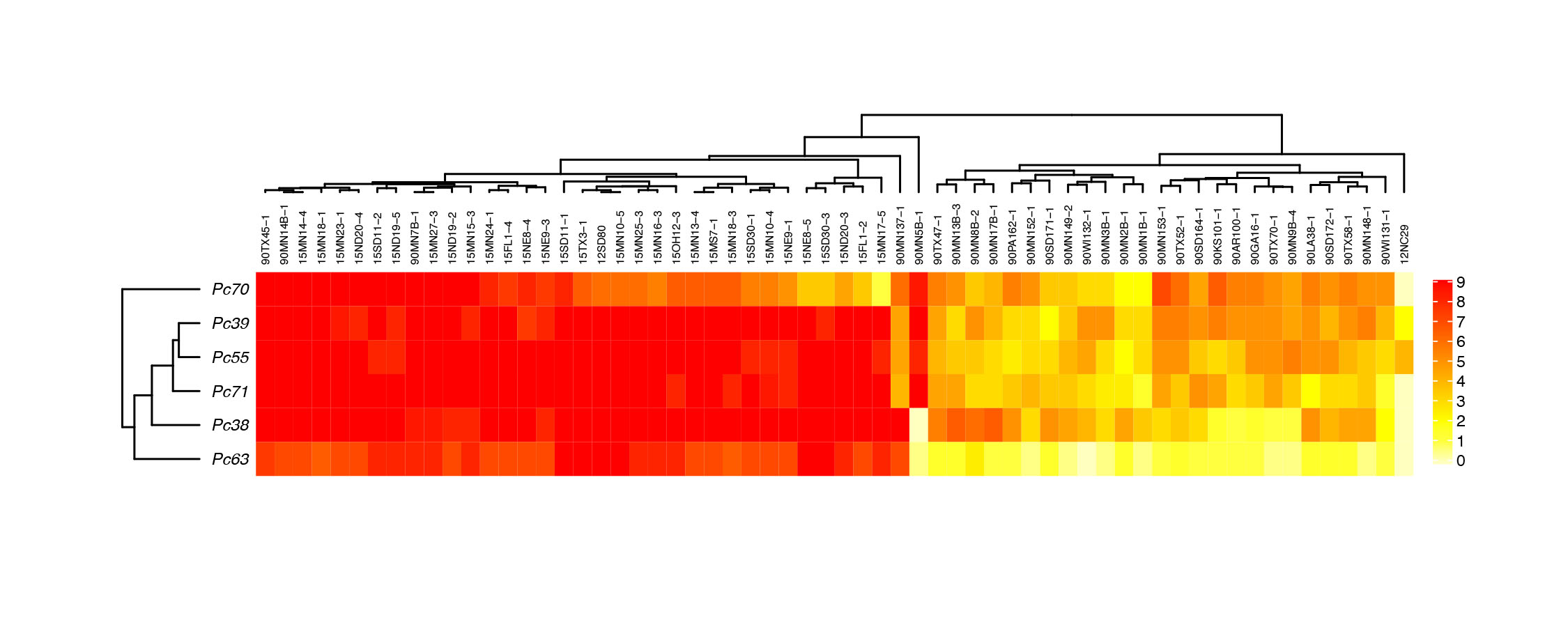

Supplement: S13 Fig — Infection scores were converted to a numeric scale (0 = resistance to 9 = susceptibility) for heatmap generation. (TIF) [file pgen.1009291.s013.tif]

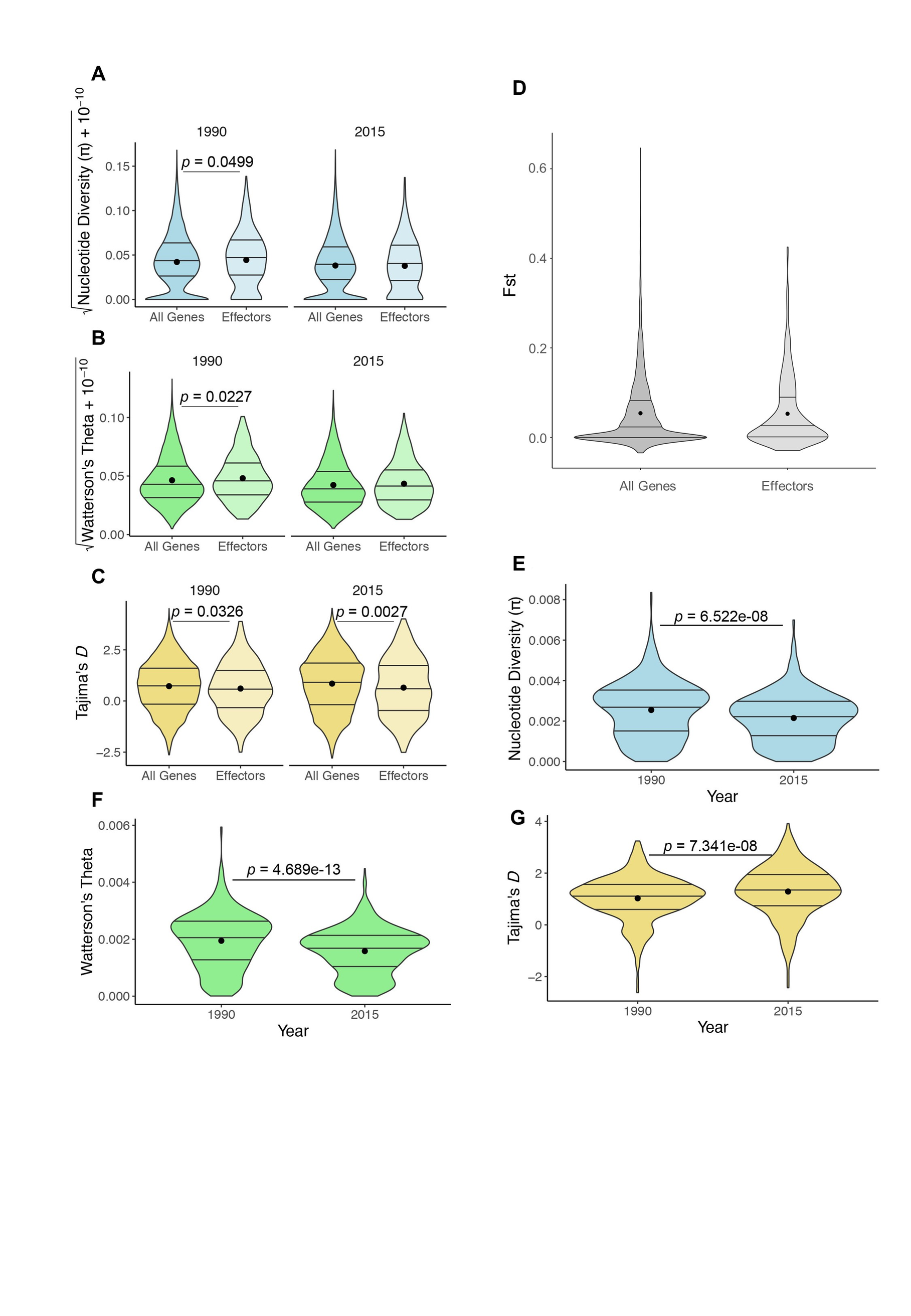

Supplement: S14 Fig — (A) Distribution of nucleotide diversity (π) values for all genes and predicted effectors of 12SD80 using variants from 1990 and 2015 populations. (B) Distribution of Watterson’s θ values for all genes and predicted effectors of 12SD80 using variants from 1990 and 2015 populations. (C) Distribution of Tajima’s D values for all genes and predicted effectors of 12SD80 using variants from 1990 and 2015 populations. (D) Distribution of Fst values for all genes and predicted effectors of 12SD80 using variants from 1990 and 2015 populations. (E) Distribution of nucleotide diversity (π) values for contigs in the reference genome 12SD80 using variants from 1990 and 2015 collections. (F) Distribution of Watterson’s θ values for contigs in the reference genome 12SD80 using variants from 1990 and 2015 collections. (G) Distribution of Tajima’s D values for contigs in the reference genome 12SD80 using variants from 1990 and 2015 collections. Values in A and B were transformed as indicated on the y-axes to aid in visualization. Lines in the violin plots represent quartiles. p-values were calculated using the Wilcoxon rank-sum test. (TIF) [file pgen.1009291.s014.tif]

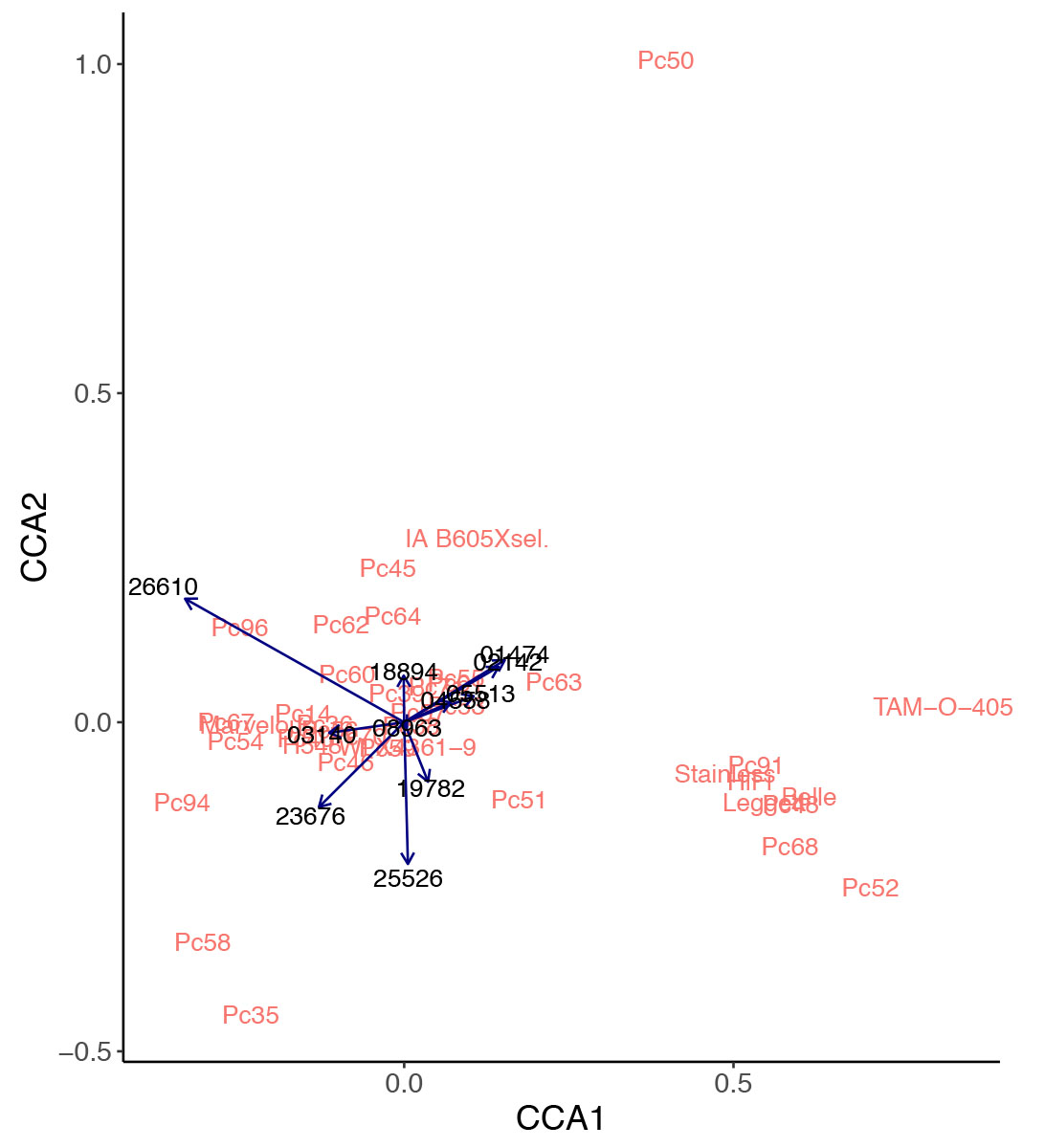

Supplement: S15 Fig — In the ordination plot, the black arrows show the contribution of the presence/absence variation of particular genes to the CCA axes. The PCASD_ prefixes were left off for clarity. The names in red are the oat lines in the differential set. CCA1 explained 44.9% of the total inertia and CCA2 explained another 15.6% of the total inertia. (TIF) [file pgen.1009291.s015.tif]

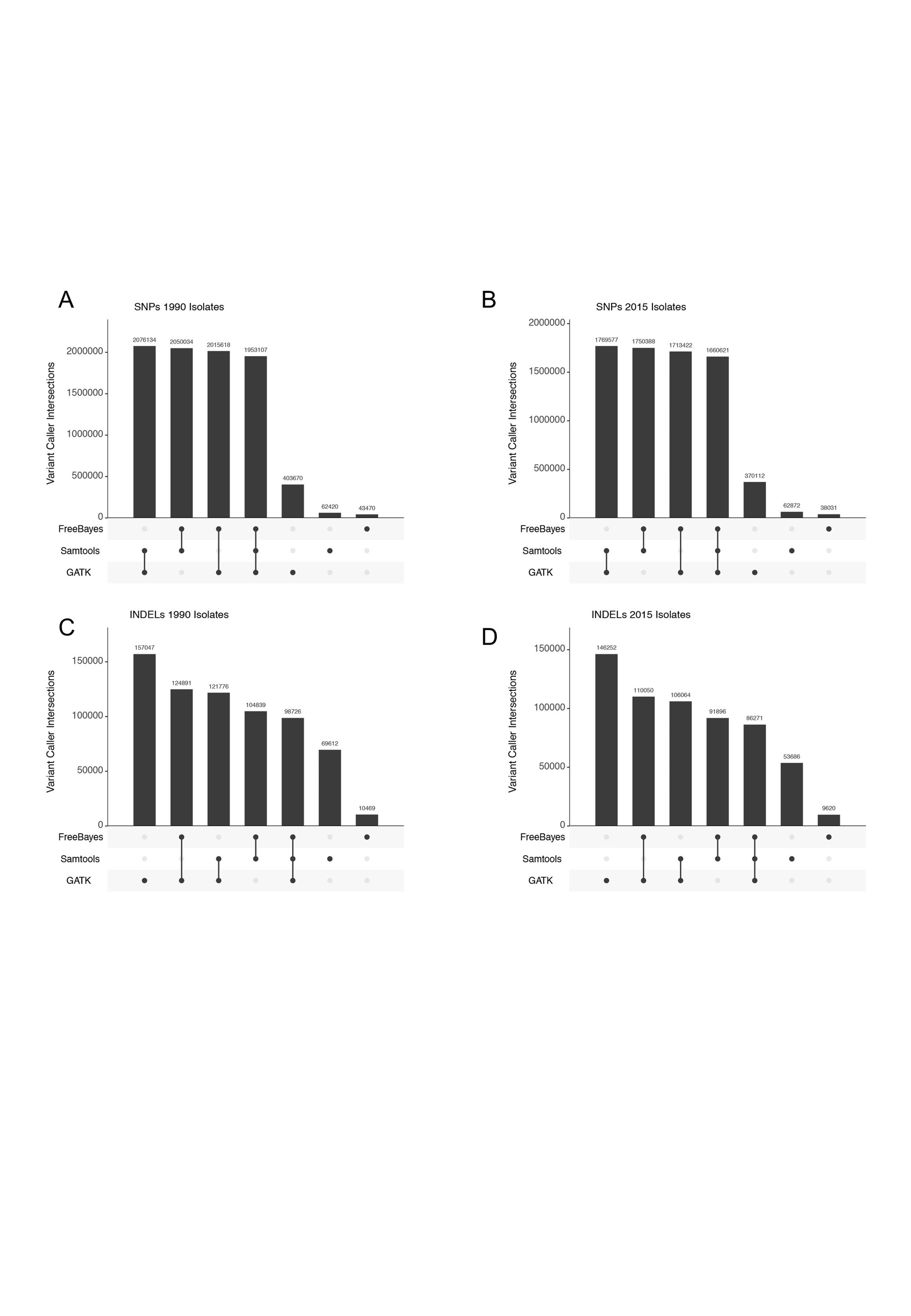

Supplement: S16 Fig — (A) SNPs unique to and shared between the three callers in the 1990 isolates. (B) SNPs unique to and shared between the three callers in the 2015 isolates. (C) INDELs unique to and shared between the three callers in 1990. (D) INDELs unique to and shared between the three callers in 2015. (TIF) [file pgen.1009291.s016.tif]

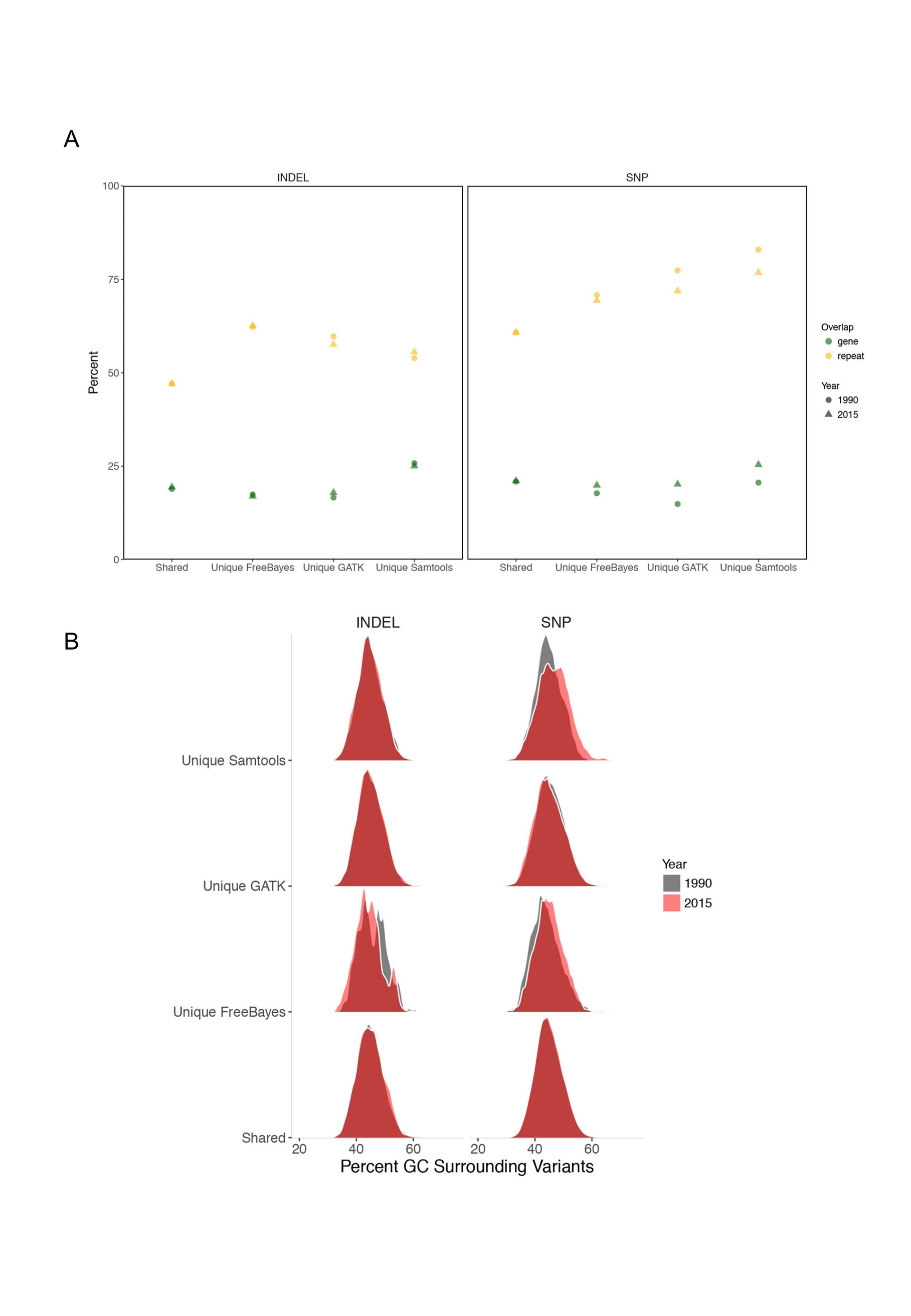

Supplement: S17 Fig — (A) Percent overlap of shared and unique variants with genes and repeats for 1990 and 2015 isolate datasets. (B) GC percent in 1 kbp windows surrounding shared and unique variants for 1990 and 2015 isolate datasets according to variant caller. (TIF) [file pgen.1009291.s017.tif]
