# Supplementary material for: Increased virulence of Puccinia coronata f. sp.avenae populations through allele frequency changes at multiple putative Avr loci
Source: PLoS Genet. 2020 Dec 28;16(12):e1009291. doi: 10.1371/journal.pgen.1009291 (PMC7793281; doi:10.1371/journal.pgen.1009291)
Supplement: S3 Table — (DOCX) [file pgen.1009291.s020.docx]

**Table S3.** Mean values of nucleotide diversity and neutrality in all genes and effectors.

|  | **1990 All Genes** | **1990 Effectors** | **2015 All Genes** | **2015 Effectors** |
| --- | --- | --- | --- | --- |
| Mean nucleotide diversity (π) | 0.0028 | 0.0029 | 0.0024 | 0.0023 |
| Wilcoxon rank sum test *p* value | 0.0499 | | 0.9763 | |
| Mean Watterson’s Theta (θ_w_) | 0.0026 | 0.0027 | 0.0022 | 0.0022 |
| Wilcoxon rank sum test *p* value | 0.0227 | | 0.1238 | |
| Mean Tajima’s D | 0.727 | 0.610 | 0.851 | 0.654 |
| Wilcoxon rank sum test *p* value | 0.0326 | | 0.0027 | |
